# Increasing the feasibility, impact, and equity of the Medicare Annual Wellness Visit (AWV) with a practice tailored AWV intervention: A stepped wedge clinical trial protocol

**DOI:** 10.1371/journal.pone.0329004

**Published:** 2025-08-08

**Authors:** Derjung M. Tarn, Wilson D. Pace, Kurt C. Stange, Chi-hong Tseng, Neil S. Wenger

**Affiliations:** 1 DARTNet Institute, Aurora, Colorado, United States of America; 2 Division of General Internal Medicine/ Health Services Research, Department of Medicine, David Geffen School of Medicine at UCLA, University of California, Los Angeles, Los Angeles, California, United States of America; 3 Department of Family Medicine, David Geffen School of Medicine at UCLA, University of California, Los Angeles, California, United States of America; 4 Center for Community Health Integration and Departments of Family Medicine & Community Health, Population & Quantitative Health Sciences, and Sociology, Case Comprehensive Cancer Center, Case Western Reserve University, Cleveland, Ohio, United States of America; Taipei Medical University, TAIWAN

## Abstract

**Background:**

Older adults vastly underutilize evidence-based preventive health services and screenings that reduce illness, morbidity and mortality. The free-to-patient Medicare Annual Wellness Visit (AWV) is an opportunity to enhance preventive healthcare use, but also is underused.

**Objectives:**

To evaluate the effect of a practice-tailored intervention on the sustained use of Medicare AWVs and on guideline-recommended preventive services and racial/ethnic disparities in 3 types of practice settings.

**Methods:**

This is a stepped wedge cluster randomized controlled trial. The intervention will be implemented at the practice level in 24 primary care practices across the country (8 community-based, 8 academic, and 8 serving medically underserved populations). Electronic health record data will be used to assess changes in AWV and preventive service delivery rates and racial, ethnic, and gender disparities. Semi-structured interviews will be conducted with clinicians/staff and patients, and clinicians will be surveyed to assess the process and acceptability of the intervention. The protocol is registered on clinicaltrials.gov (NCT05910736).

**Results:**

Analyses will determine the effect of the intervention on AWV visit and preventive health services use at 12- and 24-months post-intervention implementation. Additional analyses will evaluate the effect of the intervention on reducing racial/ethnic disparities.

**Conclusions:**

A practice-tailored intervention has the potential to increase use of AWVs and preventive health services, and reduce racial/ethnic disparities, in diverse practice settings.

## Introduction

Older adults vastly underutilize evidence-based preventive health services that are proven to reduce serious illness, morbidity and mortality. In fact, fewer than half of adults aged 65 and older are up-to-date on a core set of preventive health services (flu vaccine in the past year, pneumonia vaccine (ever), colorectal screening, and mammogram in the past 2 years (for women)). [1,2] Those at greatest risk for receiving poor preventive care include racial and ethnic minority groups and persons of low socioeconomic status. For example, colorectal cancer incidence and mortality rates are highest in Black people, [3,4] but along with Hispanic and Asian individuals, they are screened less frequently than White patients. [5–7] Black patients also complete fewer mammograms than White patients. [5] Interventions to remedy underutilization of preventive health services in older adults have mostly targeted individual preventive health services, rather than the totality of preventive services needed by patients.

The Medicare Annual Wellness Visit (AWV) represents a promising but underused opportunity to improve uptake of evidence-based screening services. This free-to-the-patient Medicare benefit gives providers dedicated time to focus on preventive healthcare and has been shown to increase use of preventive health services. Medicare reimburses two different types of preventive health visits: 1) the Initial Preventive Physical Examination (IPPE), known as the “Welcome to Medicare Preventive Visit” (a one-time benefit); and 2) the AWV (a yearly benefit for patients after their first 12 months of Medicare Part B eligibility). [8] This protocol refers to these visits collectively as AWVs, though the requisite tasks for the visits differ slightly. [8]

Patients completing an AWV are more likely than those without an AWV to complete preventive health services such as: cancer screenings (e.g., colorectal, breast and cervical); vaccinations (e.g., influenza, pneumococcal, and herpes zoster); screening about tobacco use, depression and fall risks; advance care planning; and abdominal aortic aneurysm screening. [9–19] Despite its promise, less than half of eligible fee-for-service Medicare patients completed an AWVs in 2020, [20] likely due to challenges at patient, clinician, and practice levels. [21–24] Further, racial/ethnic disparities exist, with lower AWV uptake among racial/ethnic minority patients. [25] Practices caring for underserved populations have the lowest adoption rates. [26]

The study investigators developed a novel multi-level intervention to address the complexities of increasing AWV uptake at multiple levels: patient (demand), clinician (supply of services), office/nursing staff, and organization. [27] The intervention was piloted in a private practice in Colorado as part of an NIH R61. [27] Implemented at the practice level, the ‘Practice-Tailored AWV Intervention’ (PT-AWV) couples electronic health record (EHR) modifications or tools with tailored practice redesign tools and approaches, which include materials targeted for racial/ethnic minority patients. The intervention creates practice changes that should persist over time, but data are lacking on the effect of the PT-AWV on evidence-based interventions shown to affect health, on how long these changes persist, and on how the intervention can best be tailored for different types of practice settings. This protocol describes a stepped wedge clinical trial designed to test the effect of the PT-AWV intervention on AWV and preventive health services use. We hypothesize that the intervention will increase uptake of AWVs and use of preventive health services.

## Methods

### Study objectives

The **primary** study objectives are to evaluate the effect of the PT-AWV intervention on the use of: 1) AWVs and 2) United States Preventive Services Task Force (USPSTF) and Centers for Disease Control and Prevention/ Advisory Committee on Immunization Practices (CDC/ACIP)-recommended preventive services (Table 1) in 3 different types of practice settings. **Secondary** objectives are to evaluate the effect of the PT-AWV intervention on reducing racial/ethnic disparities in AWV utilization and to evaluate the sustainability of the intervention. **Tertiary objectives** are to evaluate factors affecting implementation and sustainability of the PT-AWV intervention tools and approaches, implementation strategies, and intervention effects in diverse practice settings.

**Table 1:** Preventive health services assessed, recommended frequency and age, and evidence for use/no use^*^.

Outcome Measure	Recommended Frequency of Screening	Recommended Age for Screening	Grade^±^
Vaccinations [46]			
Influenza [47]	Yearly	6 months of age or older	N/A
Herpes zoster (shingles) [48]	Once (2 doses)	50 or older	N/A
Pneumococcal (PCV15; PCV20; PCV21, PPSV23) [49,50]	Once	65 and older regardless of previous vaccination history	N/A
Tetanus (Td or Tdap) [51]	Booster every 10 years	19 and older	N/A
Cancer screening			
Colorectal cancer [52]	Every 1–10 years depending on type of screening	45-49 years 50-75 76-85	B A C
Breast cancer [53]	Every 2 years	Women 40–74 years 75 or older	B I
Cervical cancer [54]	Every 3–5 years	Women 21–65 years Older than 65	A D
Other screening			
Osteoporosis [55]	At least once	Women aged 65 and older	B
Hepatitis C[56]	Once	18-79	B
Alcohol misuse [57]	Yearly	18 or older	B
Tobacco use [58]	Yearly	All adults	A
Depression [59]	Yearly	All adults	B
Advance care planning [60,61]			N/A

*Based on USPSTF, CDC/ACIP guidelines, will adjust as needed to match guidelines at the time of data collection

±A = recommended (high certainty of substantial net benefit); B = recommended (high certainty of moderate net benefit or moderate certainty of moderate to substantial net benefit); C = selectively offer based on professional judgement and patient preferences (moderate certainty of small net benefit); D = recommends against service (no net benefit or harms outweigh the benefits); I = current evidence is insufficient to assess balance of benefits and harms

### Design and setting

This multi-site, stepped wedge cluster randomized controlled trial will take place in 24 primary care practices around the United States (8 community-based practices, 8 academic practices, and 8 practices serving medically underserved populations). Recruitment and intervention implementation occur at the practice level. The stepped wedge cluster design is a pragmatic study design that includes an initial control period in which no practices receive the intervention. In each wedge, randomized clusters of practices cross to the intervention condition at regular intervals, and there is a period at the end of the study where all practices have been exposed to the intervention. Data are collected throughout the study so that each cluster of practices contributes to both the control and intervention periods. [28–30] The intervention will be implemented sequentially over 15 months, with 4 clusters of 6 practices each switching from control to intervention every 3 months. For each practice, intervention implementation will occur over a 6-month period.

**Fig 1:**
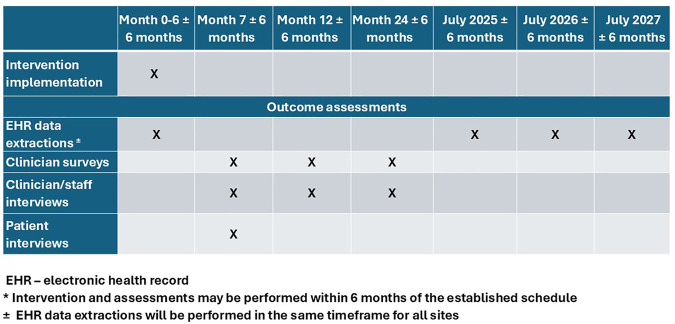
Schedule of intervention and assessments for a single practice, starting from intervention implementation*.

Primary and secondary outcomes will be assessed through EHR data extractions. In addition, we will evaluate intervention implementation (tertiary outcomes) by collecting qualitative semi-structured interview data on up to 10 patients per practice, and up to 10 clinicians/staff per practice. Clinician and staff interviews will occur immediately, 12- and 24-months post-implementation. Clinicians also will be surveyed at those time points regarding barriers and facilitators to AWV completion. Fig 1 depicts the schedule of assessments. All procedures have been approved by the University of California Institutional Review Board.

### Participant inclusion criteria

***Practices*** participating in the study must be primary care practices providing care for Medicare patients. ***Patients*** must have Medicare insurance (fee-for-service or Medicare Advantage), be aged 50 and older, and have at least 1 encounter in the past 12 months at a participating practice. In addition to assessing statistical outcomes on a population cohort of practice patients, we will conduct semi-structured interviews with patients meeting inclusion criteria for the population cohort and clinicians/staff working at participating practices who conduct, participate, schedule, follow-up on, or supervise AWV-related procedures.

### Randomization of practices

The study statistician will use stratified randomization to assign the order and timing of intervention implementation in practices. Stratification will be by organization to ensure that each cluster contains two practices from each organization. Randomization within each strata will be by simple randomization. [31] Randomization will be performed before any practices are engaged in intervention implementation activities. Blinding procedures are not applicable for this study.

### Intervention/ processes

The practice redesign tools and approaches of the PT-AWV intervention (Table 2) are based on principles consistent with the Practice Change and Development Model, [32,33] which attempts to transform culture and thinking to yield sustained changes in practice. Initial practice assessments will be conducted with a practice champion identified by the practice, who will work with the investigative team to champion the intervention and its continued implementation and use, and to ensure completion of all study assessments. The initial assessments will yield information about the practice’s motivation, resources, facilitators to conducting AWVs, current practice workflows and templates for AWVs, preferred workflows, and opportunities for change. Assessments will be performed via video conferencing, which proved effective and efficient for intervention implementation and data collection in our prior work. [27]

**Table 2:** Crosswalk between elements of the model for practice change and development, intervention tools/approaches to address barriers to AWVs, and options to tailor intervention.

Model Element and Proposed Operationalization/Assessments	Tools/ Approaches to address identified barriers to AWV use	Options for Tailoring
**Motivation of key stakeholders** Identify people in the practice with capacity to help realize change and conduct AWVs Assess individual motivation for conducting AWVs Assess alignment of motivation of key stakeholders for conducting AWVs Assess how motivation may result in change in practice regarding AWVs	Identify practice champion Training sessions, materials, feedback on: AWV benefits, terminology potential for increased revenue billing strategies AWV documentation requirements practice workflow around performing and scheduling AWVs; role-specific tasks Increase duration of visits for AWVs	Trainings can be individual, in group sessions, or both Frequency and modes (e.g., email, text messaging) of dissemination of written materials may vary Opportunity for physicians to use data for Maintenance of Certification practice improvement activity
**Resources for change** Identify current approach and culture regarding AWVs, focusing on specific processes and workflows for when, where, how, and by whom AWVs are currently delivered Identify resources that enhance practices’ ability to provide AWVs, availability of dedicated staff to promote AWVs Assess ‘connectedness’ of agents – leadership, flexibility, history of change, EHR capability	Workflow redesign Identify provider(s) to perform AWVs (physician, physician assistant, nurse practitioner, other) EHR-based notifications Point-of-service notifications; use read-write real-time clinical decision support to inform preventive services needed Training sessions, materials, feedback on using the EHR-based tools Incorporate provider and staff use of notifications into workflow AWV templates to guide documentation Define strategies to complete time-consuming HRA	Workflows will be tailored to practice needs and resources Type of provider(s) selected to perform AWVs may differ Practices with a HL7 Fast Healthcare Interoperability Resources (FHIR) server may choose to receive EHR notifications Approaches to completing HRA before the visit Send form to patients via email, postal mail, EHR portal Staff to complete by phone Patients asked to show up extra early for AWV
**Outside motivators** Identify forces that shape how the practice sees its opportunities for conducting AWVs Develop ways to address barriers	Monthly practice feedback reports on AWV/ preventive health services use and opportunities for improvement Use of a patient registry to identify those without an AWV in the past 12 months and invite them for AWVs Optional study practice meetings for all participating practices to join and share obstacles and successful strategies	Reports tailored to compare providers to other providers in their practice (either de-identified or identifiable), or to others in the study Invite patient for AWVs by phone, text message, or via patient EHR portal
**Opportunities for change** Identify practice members’ perceived opportunities for conducting more AWVs Identify and share opportunities that the practice may not see	Recommend AWVs to patients Patient education about importance of AWVs and preventive care Provider and staff training sessions, materials, feedback on how to message patients about AWVs and answer frequently asked patient questions Tailored information about preventive screenings Personalized health plan Patient notifications about the need for an AWV/ preventive services Patient reminders 4 weeks after services are ordered (if service not completed)	Workflow will designate practice member(s) to recommend/ schedule patients for AWVs when they present for other types of office visits Materials may be available in waiting room, handed to patient by office staff or provider, mailed to those on registry Information about preventive screenings will be tailored by patient race/ethnicity Tailor personalized health plan to convey patient use of services Notifications and reminders may be sent via text message, EHR patient portal, or postal mail

AWV-annual wellness visit, EHR-electronic health record, HRA-Health Risk Assessment

The assessments will be used to customize intervention components based on practices’ unique characteristics, including staff workflow, resources, and EHR capability. The exact content of the intervention will be tailored based on each practice’s individual needs. Intervention components include practice redesign tools and approaches, and EHR tools.

Implementation of the PT-AWV intervention will take place at the practice level over 6 months. Intervention implementation will include “turning on” selected EHR-based tools, providing access to selected documentation and other templates, and training practice members on the use of these tools. Provider and staff training sessions will touch on desired practice workflows for conducting AWVs, benefits of AWVs, documentation requirements, services that clinicians can perform with the AWV without incurring extra patient cost, and potential billing strategies. The research team will interface with the practice champion regularly during the 6-month implementation period to identify and remedy barriers to conducting AWVs. Practices will receive feedback reports on their AWV and preventive health services completion rates starting about 4–6 months after they begin intervention implementation until 12 months post-implementation. Practices will determine the desired frequency of these reports, which can be provided monthly to quarterly. Concomitant interventions occurring during the trial will be tracked.

### Study outcomes

***Primary study outcomes*** are**:** *1) AWV utilization*; assessed by examining: the % of eligible patients completing an AWV (CPT codes G0438, G0439, G0468) or IPPE (CPT code G0422) in the past 12 months and *2) Completion of recommended preventive health services* (measured using a composite preventive health services score depicting the % of total recommended preventive health services that are up to date of a maximum of 12 recommended services per patient; measured on the patient-level. As an example of the second primary study outcome, a male patient has a maximum of 9 recommended services because cervical cancer screening, breast cancer screening and osteoporosis screening are not routinely recommended; a female with a history of an allergic reaction to the influenza vaccine would have a maximum of 11 recommended services. AWV utilization will be compared for our secondary objective of evaluating the effect of the intervention on racial/ethnic disparities in utilization. ***Secondary outcomes*** are utilization of individual preventive health services *(*% of patients up to date for individual preventive health services endorsed by the USPSTF and CDC/ACIP (Table 1**).** Primary and secondary outcomes will be assessed at 12 and 24 months after intervention implementation. ***Tertiary outcomes*** (evaluated for tertiary study objective) are facilitators and barriers to intervention adoption and implementation. These outcomes will be collected from interviews and meetings with patients, clinicians, and staff, and from clinician surveys.

### Sample size calculations

We used data from a previous study [27] to inform sample size calculations. In our previous study, the PT-AWV intervention increased AWVs from 8% at baseline to 54% eight months post-intervention in AWV-eligible patients (n = 213, 576 and 639 patients at baseline in 3 practices). Behaviors of practices recruited from the same healthcare organizations may be correlated, so we will adjust power analyses using intra-class correlation (ICC). Based on our preliminary data, intra-class correlation for site was 0.03 and within subject correlation was −0.003 for AWV use one year apart. We conservatively estimate ICCs from.03 to.05 in our calculations. Clinically meaningful increases would conservatively be 10% for all outcomes. With simulations based on linear mixed effects models, six hundred patients per practice would yield over 99% power to detect a 10% increase in AWVs for baseline AWV rates of 10–50% and in the composite preventive health services score (type-I error rate α = 0.05 and ICC = 0.03 and 0.05). We estimate our sample size for this project to be a mean of 700 patients per practice.

### Data management plan

Data use agreements have been established with all participating practices for the transfer of limited data. The data coordinating center will receive or extract data from practices and process it through a HIPAA-compliant storage and file transfer system. They will clean and standardize the data, perform data quality checks, and transfer limited analytic datasets to UCLA for analysis. Technical safeguards will be in place to protect storage and transmission, including end-to-end encryption and firewall protection.

### Data monitoring/ safety considerations

Safety oversight will be under the direction of a Data and Safety Monitoring Board (DSMB). Members of the DSMB are independent from the sponsor and from study conduct and have no conflicts of interest. The Board consist of a statistician, health services researcher, and informaticist. The DSMB will meet at least annually to assess study progress, conduct, and safety data. Adverse events are not anticipated because the intervention is performed at the practice level, and not with patients. The risks of the study are minimal as the intervention promotes practices that are part of routine clinical care. Protocol amendments will be communicated to the DSMB during routine reporting.

### Statistical analyses

Descriptive statistics will be used to describe demographics of the primary patient cohort. For each practice and organization, we will summarize use of AWVs and preventive health services at baseline and every 6 months up to the end of the study (24 months after the completion of intervention implementation in the last set of practices). We also will use run charts to illustrate the monthly percentage of eligible patients who utilized AWVs and each preventive health service assessed (Table 1), as well as a composite preventive health services score.

Linear mixed effects models [34–38] will be used to assess the effect of the intervention on the rates of use of AWVs (primary outcome), on the composite preventive health services score (primary outcome; continuous variable), and on the rates of use of individual preventive health services (secondary outcome). Intervention effects will be assessed by comparing rates at baseline, 12 months, and 24 months using a categorical time variable (baseline, 12 months, and 24 months). We will include covariates when comparing the change in AWV and preventive services use: age, gender, race (Asian, Black/African-American, White, Other), ethnicity (Hispanic, non-Hispanic), insurance coverage (fee-for-service Medicare, Medicare Advantage), and number of office visits per year. We will also include practice setting (community-based, FQHC, academic), practice size, and percentage of racial/ethnic minority patients. These models account for correlation between repeated observations in the same cluster of practices and the effects of time and treatment. Piece-wise constant time effect will be considered to accommodate possible non-linear time trends. These models will accommodate correlations within practices. All analyses will be carried out based on the intention to treat principle. Sensitivity analyses will examine whether the treatment effects depend on exposure time by including a random effect in the models to represent varying treatment effects by exposure time. [39]

Subgroup analyses will examine heterogeneity of treatment effects by patient and practice characteristics, such as patient race/ethnicity, number of chronic conditions, and practice type (community-based, academic, underserved) on the effect of the intervention. An interaction term between the categorical treatment time variable (baseline, 12 months, and 24 months) and the variable of patient or practice characteristic will be included in the model for this analysis.

A secondary study objective is to examine the effect of the intervention on reducing racial/ethnic disparities in AWV utilization. For each practice and organization, we will summarize use of individual preventive health services by patient race/ethnicity at baseline and every 6 months (up to month 48 of the study) using descriptive statistics. We will also use run charts to illustrate and compare monthly AWV utilization by patient race/ethnicity. Linear mixed effects models will be used to directly estimate the percentage change in AWVs by race/ethnicity. [34–38] These models will include fixed effects of treatment, time, treatment-race/ethnicity interaction and patient and practice-level covariates. Patients and practices are considered as random effects and piece-wise constant time effect will be constructed to compare the racial/ethnicity disparities in AWV use before and after the intervention, based on the estimates of fixed effects.

Mixed effects models accommodate the imbalance of patient data, assuming that they are missing at random. Based on our preliminary data, we expect few missing data for patient age, gender, insurance and number of office visits per year. For missing data on race, we will perform analyses with missing race as a variable.

### Semi-structured interview and survey analyses

The study will evaluate intervention adoption, implementation and sustainability (tertiary objectives) by describing facilitators and barriers to adoption through analysis of semi-structured interviews and clinician surveys. Members of the research team will analyze meeting notes and semi-structured interviews. Two members of the team will review transcripts, both independently and together, to inductively generate codes relevant to pre-defined measures guided by the RE-AIM framework. [40–43] Co-investigators will participate in selected analyses to ensure the trustworthiness and robustness of the findings. We will use a template coding approach [44] to efficiently identify and segment data relevant to pre-defined measures guided by the RE-AIM framework. Case-based matrices [45] will help us to refine and synthesize our understanding of modifiable reasons that emerge as obstacles for implementing and sustaining the PT-AWV intervention, strategies for mitigating obstacles, and practice strengths that facilitate implementation and sustainability. Analyses will compare and contrast themes raised in different practice settings. Analyses will be done in ATLAS.ti.

Clinician survey responses will be tabulated to determine the toolkit components being used, major barriers to doing AWVs, and facilitators of the visits. The investigative team will use content analysis to analyze free-text responses to survey questions on barriers and facilitators of AWV completion.

### Ethical considerations and declarations

This protocol was approved by the University of California, Los Angeles Institutional Review Board on June 28, 2023 (IRB #23–000679).

***Population cohort.*** A waiver of informed consent was obtained for data extractions to assess outcome measures for the population cohort, as it is not feasible to obtain informed consent for all patients in this cohort. The investigators will receive only limited datasets for analyses.

***Semi-structured interview and clinician survey cohorts***. A waiver of signed informed consent was obtained for semi-structured interviews and clinician surveys. These procedures are low risk and written informed consent would be difficult to obtain as the assessments do not occur in-person. Informed consent for interviews and surveys will be provided by giving participants a study information sheet or (for interviews) obtaining verbal consent.

### Status and timeline of study

**Fig 2:**
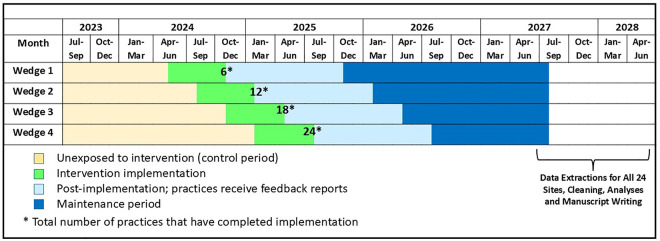
Stepped Wedge Study Design and Timeline.

The study timeline is depicted in **Fig 2**. At the time of this protocol submission, intervention implementation has been initiated for practices in the first 3 wedges of the study. Preliminary data extractions are in progress. Data extractions for 12- and 24-month post-intervention outcomes assessment are estimated in August 2026 and August 2027, with corresponding results expected in August 2027 and August 2028.

## Discussion

This project will assess a multilevel intervention to increase AWVs, which in turn is hypothesized to improve use of preventive services and the equity of care in diverse practice settings. We expect this pragmatic trial to demonstrate the effectiveness of the PT-AWV intervention for increasing the use of AWVs and USPSTF and CDC/ACIP-recommended preventive health services across practices, and to reduce racial/ethnic disparities in AWV use. Information on successful strategies for implementing the PT-AWV intervention and for overcoming obstacles to implementation in different practice settings will be used to further refine the intervention for broader dissemination. Trial results will be disseminated via conference presentations and publications authored by the study investigators. Authorship on manuscripts will follow guidelines established by the International Committee of Medical Journal Editors (ICMJE).

Several study limitations exist. First, baseline AWV rates in practices may increase over time, but practices will still benefit from a systematic multi-level approach to promoting AWVs. We will adjust our analyses for time trends to remove the secular time trend. Second, some organizations may already have EHR tools similar to those developed in the proposal, thus reducing the effect of the intervention. Our previous experiences indicate that provision of EHR tools alone is insufficient, and that practices need help to maximize their use of and to refine these tools. Third, potential contamination may occur since healthcare organizations may contribute multiple practices to the study and personnel may shift across practices. This is unlikely to have a large effect since our ICCs were low. Fourth, we are not audio recording office visits so will not know whether or how providers discuss preventive health services. This limits our ability to understand the mechanism of preventive care promotion and the level of provider influence on patient completion of cancer screenings and vaccinations.

In conclusion, this study’s anticipated increases in patient use of Medicare AWVs are expected to result in increased fulfillment of preventive health services, which in turn can improve population health and lower mortality.

## Supporting information

S1 Protocol AWV R01 protocol. (DOCX)

S2 checklist SPIRIT Checklist. (PDF)
