# Supplementary material for: Increasing the feasibility, impact, and equity of the Medicare Annual Wellness Visit (AWV) with a practice tailored AWV intervention: A stepped wedge clinical trial protocol
Source: PLoS One. 2025 Aug 8;20(8):e0329004. doi: 10.1371/journal.pone.0329004 (PMC12333998; doi:10.1371/journal.pone.0329004)
Supplement: S1 Protocol — (DOCX) [file pone.0329004.s001.docx]

**Abbreviated Title: AWV R01 Protocol**

**UCLA IRB #:** 23-000679

**Version Date:** 08 August 2023

**Increasing the Feasibility, Impact, and Equity of the Medicare Annual Wellness Visit (AWV)**

**National Clinical Trial (NCT) Identified Number: NCT05910736**

**Principal Investigator: Derjung Mimi Tarn, MD, PhD**

**Department of Family Medicine**

**University of California, Los Angeles**

[dtarn@mednet.ucla.edu](mailto:dtarn@mednet.ucla.edu)

**Grant Number: 1R01AG081996**

**Funded by:**  **NATIONAL INSTITUTE ON AGING**

**Version Number: 1.0**

**August 8, 2023**

## TABLE OF CONTENTS

[TABLE OF CONTENTS 2](#_Toc141435164)

[STATEMENT OF COMPLIANCE 6](#_Toc141435165)

[1 PROTOCOL SUMMARY 7](#_Toc141435166)

[1.1 Synopsis 7](#_Toc141435167)

[1.2 Schema 9](#_Toc141435168)

[1.3 Schedule of Activities (SOA) 9](#_Toc141435169)

[2 STUDY TEAM 10](#_Toc141435170)

[3 INTRODUCTION 13](#_Toc141435171)

[3.1 Study Rationale 13](#_Toc141435172)

[3.2 Background 13](#_Toc141435173)

[3.3 Risk/Benefit Assessment 16](#_Toc141435174)

[3.3.1 Known Potential Risks 16](#_Toc141435175)

[3.3.2 Known Potential Benefits 17](#_Toc141435176)

[3.3.3 Assessment of Potential Risks and Benefits 17](#_Toc141435177)

[4 OBJECTIVES AND ENDPOINTS 18](#_Toc141435178)

[5 STUDY DESIGN 20](#_Toc141435179)

[5.1 Overall Design 20](#_Toc141435180)

[5.2 Scientific Rationale for Study Design 20](#_Toc141435181)

[5.3 Justification for Intervention 21](#_Toc141435182)

[5.4 End-Of-Study Definition 22](#_Toc141435183)

[6 STUDY POPULATION 22](#_Toc141435184)

[6.1 Inclusion Criteria 22](#_Toc141435185)

[6.2 Exclusion Criteria 22](#_Toc141435186)

[6.3 Inclusion of Vulnerable Participants 22](#_Toc141435187)

[6.3.1 Participation of NIH Staff or family members of study team members 22](#_Toc141435188)

[6.4 Inclusion of Pregnant Women, fetuses or neonates 23](#_Toc141435189)

[6.5 Lifestyle Considerations 23](#_Toc141435190)

[6.6 Screen Failures 23](#_Toc141435191)

[6.7 Strategies for Recruitment and Retention 23](#_Toc141435192)

[6.7.1 Costs 24](#_Toc141435193)

[6.7.2 Compensation 24](#_Toc141435194)

[7 STUDY INTERVENTION(S) OR EXPERIMENTAL MANIPULATION(S) 24](#_Toc141435195)

[7.1 Study Interventions(s) or Experimental Manipulations(s) Administration 24](#_Toc141435196)

[7.1.1 Study Intervention or Experimental Manipulation Description 24](#_Toc141435197)

[7.1.2 Administration 28](#_Toc141435198)

[7.2 Fidelity 29](#_Toc141435199)

[7.2.1 Interventionist Training and Tracking 29](#_Toc141435200)

[7.3 Measures to Minimize Bias: Randomization and Blinding 29](#_Toc141435201)

[7.4 Study Intervention/Experimental Manipulation Adherence 29](#_Toc141435202)

[7.5 Concomitant Therapy 30](#_Toc141435203)

[7.5.1 Rescue Therapy 30](#_Toc141435204)

[8 STUDY INTERVENTION/EXPERIMENTAL MANIPULATION DISCONTINUATION AND PARTICIPANT DISCONTINUATION/ WITHDRAWAL 30](#_Toc141435205)

[8.1 Discontinuation of Study Intervention/Experimental Manipulation 30](#_Toc141435206)

[8.2 Participant Discontinuation/Withdrawal from the Study 30](#_Toc141435207)

[8.3 Lost to Follow-up 30](#_Toc141435208)

[9 STUDY ASSESSMENTS AND PROCEDURES 30](#_Toc141435209)

[9.1 Screening Procedures 30](#_Toc141435210)

[9.1.1 Screening activities performed prior to obtaining informed consent 30](#_Toc141435211)

[9.1.2 Screening activities performed after a consent for screening has been obtained 31](#_Toc141435212)

[9.2 Study Evaluations & Procedures 31](#_Toc141435213)

[9.2.1 Biospecimen Evaluations 31](#_Toc141435214)

[9.2.2 Samples for Genetic/Genomic Analysis 31](#_Toc141435215)

[9.3 Safety Assessments 32](#_Toc141435216)

[9.4 Adverse Events and Serious Adverse Events 32](#_Toc141435217)

[9.4.1 Definition of Adverse Event 32](#_Toc141435218)

[9.4.2 Definition of Serious Adverse Events (SAE) 32](#_Toc141435219)

[9.4.3 Classification of an Adverse Event 33](#_Toc141435220)

[9.4.4 Time Period and Frequency for Event Assessment and Follow-Up 34](#_Toc141435221)

[9.4.5 Adverse Event Reporting 35](#_Toc141435222)

[9.4.6 Serious Adverse Event Reporting 35](#_Toc141435223)

[9.4.7 Events of Special Interest 35](#_Toc141435224)

[9.4.8 Reporting of Pregnancy 36](#_Toc141435225)

[9.5 Unanticipated Problems 36](#_Toc141435226)

[9.5.1 Definition of Unanticipated Problems (UP) 36](#_Toc141435227)

[9.5.2 Unanticipated Problem Reporting 36](#_Toc141435228)

[10 STATISTICAL CONSIDERATIONS 36](#_Toc141435229)

[10.1 Statistical Hypothesis 36](#_Toc141435230)

[10.2 Sample Size Determination 37](#_Toc141435231)

[10.3 Populations for Analyses 39](#_Toc141435232)

[10.4 Statistical Analyses 40](#_Toc141435233)

[10.4.1 General Approach 40](#_Toc141435234)

[10.4.2 Analysis of the Primary Endpoints 40](#_Toc141435235)

[10.4.3 Analysis of the Secondary Endpoints 42](#_Toc141435236)

[10.4.4 Analysis of the Tertiary Endpoints 43](#_Toc141435237)

[10.4.5 Safety Analyses 43](#_Toc141435238)

[10.4.6 Baseline Descriptive Statistics 43](#_Toc141435239)

[10.4.7 Planned Interim Analyses 44](#_Toc141435240)

[10.4.8 Sub-Group Analyses 44](#_Toc141435241)

[10.4.9 Tabulation of individual Participant Data 44](#_Toc141435242)

[10.4.10 Exploratory Analyses 44](#_Toc141435243)

[11 REGULATORY, ETHICAL, AND STUDY OVERSIGHT CONSIDERATIONS 44](#_Toc141435244)

[11.1 Informed Consent Process 44](#_Toc141435245)

[11.1.1 Consent/Assent Procedures and Documentation 44](#_Toc141435246)

[11.1.2 Consent for minors when they reach the age of majority 45](#_Toc141435247)

[11.1.3 Considerations for Consent of NIH staff, or family members of study team members 45](#_Toc141435248)

[11.1.4 Consent of Subjects who are, or become, decisionally impaired 45](#_Toc141435249)

[11.2 Study Discontinuation and Closure 45](#_Toc141435250)

[11.3 Confidentiality and Privacy 46](#_Toc141435251)

[11.3.1 Measures Taken to Ensure Confidentiality of Data Shared per the NIH Data Sharing Policies 47](#_Toc141435252)

[11.3.2 Certificate of Confidentiality 47](#_Toc141435253)

[11.4 Future use of Stored Specimens and Data 47](#_Toc141435254)

[11.5 Safety Oversight 47](#_Toc141435255)

[11.6 Clinical Monitoring 48](#_Toc141435256)

[11.7 Quality Assurance and Quality Control 48](#_Toc141435257)

[11.8 Data Handling and Record Keeping 48](#_Toc141435258)

[11.8.1 Data Collection and Management Responsibilities 48](#_Toc141435259)

[11.8.2 Study Records Retention 50](#_Toc141435260)

[11.9 Protocol Deviations and Non-Compliance 50](#_Toc141435261)

[11.9.1 NIH Definition of Protocol Deviation 51](#_Toc141435262)

[11.10 Publication and Data Sharing Policy 51](#_Toc141435263)

[11.10.1 Human Data Sharing Plan 51](#_Toc141435264)

[11.10.2 Genomic Data Sharing Compliance 51](#_Toc141435265)

[11.11 Collaborative Agreements 51](#_Toc141435266)

[11.11.1 Agreement Type 51](#_Toc141435267)

[11.12 Conflict of Interest Policy 51](#_Toc141435268)

[12 ABBREVIATIONS 52](#_Toc141435269)

[13 APPENDIX 54](#_Toc141435270)

[REFERENCES 59](#_Toc141435271)

## STATEMENT OF COMPLIANCE

The trial will be carried out in accordance with International Conference on Harmonisation Good Clinical Practice (ICH GCP) and the following:

- - United States (US) Code of Federal Regulations (CFR) applicable to clinical studies (45 CFR Part 46, 21 CFR Part 50, 21 CFR Part 56, 21 CFR Part 312, and/or 21 CFR Part 812)

National Institutes of Health (NIH)-funded investigators and clinical trial site staff who are responsible for the conduct, management, or oversight of NIH-funded clinical trials have completed Human Subjects Protection and ICH GCP Training.

The protocol, informed consent form(s), recruitment materials, and all participant materials have been submitted to the University of California, Los Angeles Institutional Review Board (IRB) for review and approval. Approval of both the protocol and the consent form must be obtained before any participant is enrolled. Any amendment to the protocol will require review and approval by the IRB before the changes are implemented to the study. In addition, all changes to the consent form will be IRB-approved; an IRB determination will be made regarding whether a new consent needs to be obtained from participants who provided consent, using a previously approved consent form.

## 1 PROTOCOL SUMMARY

### 1.1 Synopsis

| **Title:** | Increasing the Feasibility, Impact, and Equity of the Medicare Annual Wellness Visit (AWV) |
| --- | --- |
| **Study Description:** | The overall goal of this study is to assess the sustainability of the effect of the ***Practice-Tailored AWV (PT-AWV) intervention*** and to evaluate its impact on reducing racial/ethnic disparities in preventive healthcare. This is a stepped wedge cluster randomized controlled trial. The intervention will be implemented at the practice level in 3 major types of primary care practices across the country. A total of 24 practices will participate (8 community-based, 8 academic, and 8 safety net practices). Electronic health record (EHR) data extractions will be used to collect outcomes in a *population cohort* of patients. Semi-structured interviews will be conducted with clinicians/staff and patients to assess intervention implementation. Clinicians will also be surveyed. We hypothesize that the intervention implementation will increase AWV visit use and consequently, increase use of preventive health services at 12 and 24 months post-intervention implementation, and will reduce racial/ethnic disparities in AWV and preventive health services use. |
| **Objectives:** | ***Primary Objectives*:**  Evaluate the effect of ***the intervention*** (PT-AWV) on the use of:  1) AWVs, and  2) USPSTF and CDC/ACIP-recommended preventive services  in 3 different types of practice settings. |
|  | ***Secondary Objectives*:**  Evaluate the effect of ***the intervention*** (PT-AWV) on reducing racial/ethnic disparities in AWV utilization.  ***Tertiary Objectives:***  Evaluate factors affecting implementation and sustainability of the PT-AWV intervention tools and approaches, implementation strategies, and intervention effect in diverse practice settings. |
| **Endpoints:** | ***Primary Endpoints***:  1) AWV utilization (% of eligible patients completing an AWV or Initial Preventive Physical Examination (IPPE) in the past 12 months).  2) Composite preventive health services score (% of total recommended preventive health services that are up to date).  ***Secondary Endpoints***:  Utilization of individual preventive health services: % of patients up to date on individual preventive health services endorsed by the USPSTF and CDC/ACIP.  Primary and secondary endpoints will be assessed at 12, 18 and 24 months after intervention implementation. |
| **Study Population:** | ***Practices***: Care for patients aged 50 and older with Medicare insurance  ***Patients***: 1) aged 50 and older; 2) have Medicare insurance; 3) have had at least 1 encounter in the past 12 months at a participating practice site; and 4) are not deceased. Estimated sample size: 37,000 patients. |
| **Description of Sites/Facilities Enrolling Participants:** | N/A |
| **Description of Study Intervention:** | The PT-AWV intervention combines the use of EHR-generated tools with practice redesign tools and approaches. These tools are designed for practices and clinicians. ***EHR-generated tools*** include a registry or list of all eligible patients in a practice who need AWVs, as well as information about the preventive health services needed by each patient. There is also capability to message patients to let them know about the need for an AWV and/or preventive health services. Organizations may choose to incorporate and/or develop site-specific EHR tools to deliver documentation templates, such as SmartSets or PowerPlans (like an order set but can include documentation tools and templates).  ***Practice redesign tools and approaches*** involve designing workflows to help practices use EHR tools, training all members of practices on how to use the workflows, and training clinicians, medical assistants, and office staff regarding the tasks that need completion when patients arrive for an AWV. Resources include templates for required AWV components such as health risk assessments and personalized prevention plans, patient messaging about AWVs, clinician documentation templates, and patient-targeted educational materials about the need for different preventive health services. Feedback reports on completion of AWVs and preventive health services will be given to practices monthly or quarterly, based on practice preference.  The study team will work with practices to implement practice redesign tools during a 6-month implementation period. Training sessions will be performed via a video-conferencing platform and/or in person. |
| **Study Duration:** | 39 months |
| **Participant Duration:** | Semi-structured interviews with patients, clinicians and staff will last about 30-60 minutes. Clinician surveys will take about 5-10 minutes. Data extractions for outcome assessments will not require direct participant involvement. |

### 1.2 Schema

A total of 24 practices within 3 or more organizations will participate in this study (8 community-based, 8 academic, and 8 safety net practices). This is a stepped wedge study, in which 6 practices (no more than 2 from a single organization) will receive the intervention in each step. **Figure 1** depicts the study design and timeline.

**Figure 1. Study design and timeline.**


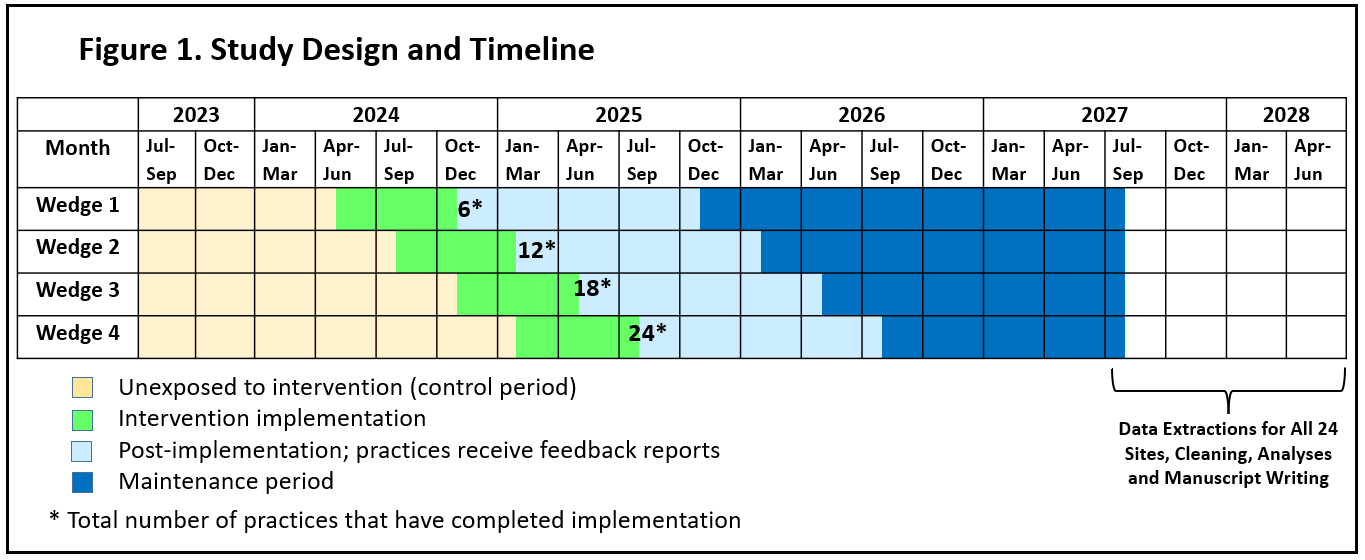


### 1.3 Schedule of Activities (SOA)

Major activities for this project **(Table 1)** include:

- Intervention implementation (see [**Section 1.2**](#_1.2_Schema) for timeline of practice participation).
- EHR data extractions (for population-level outcomes assessment).
- Semi-structured interviews with key informants (clinicians/staff) and patients (for evaluation of intervention adoption and implementation). Clinicians/staff will participate in up to 3 sets of interviews. Interviews are not longitudinal and different clinicians/staff may participate at different time points. Patients will participate in a single interview.
- Clinician surveys (for evaluation of intervention adoption).
- Generation of monthly or quarterly feedback reports for each site, based on practice preference.

| **Table 1. Schedule of evaluations for a single practice, starting from intervention implementation** | | | | | | | |
| --- | --- | --- | --- | --- | --- | --- | --- |
| **Evaluation** | **Intervention Implementation Period** | **Post-Implementation Period** | | | | | |
|  | Month 0-6 ± 6 months | Month 7 ± 6 months | Month 12 ± 6 months | Month 24 ± 6 months | July 2025 ± 6 months* | July 2026 ± 6 months* | July 2027 ± 6 months* |
| **Outcome evaluation (population-level)** | | | | | | | |
| EHR data extractions | X |  |  |  | X | X | X |
| **Semi-structured interviews / surveys** | | | | | | | |
| Clinician  surveys |  | X | X | X |  |  |  |
| Clinician/staff  interviews |  | X | X | X |  |  |  |
| Patient  interviews |  | X |  |  |  |  |  |
| **Feedback Reports** | Monthly or Quarterly (based on practice preference) | | |  | | | |
| * EHR data extractions will be performed in the same timeframe for all sites | | | | | | | |

## 2 STUDY TEAM

**Principal Investigator: Derjung Mimi Tarn, MD, PhD**

Department of Family Medicine

University of California, Los Angeles

**Co-Investigators Elizabeth Callen, PhD**

American Academy of Family Physicians National Research

Network (AAFP NRN) (operated by the DARTNet Institute)

**Wilson Pace, MD**

DARTNet Institute

**Kurt Stange, MD, PhD**

Center for Community Health Integration and Departments

of Family Medicine & Community Health, Population & Quantitative Health Sciences, Oncology, and Sociology

Case Western Reserve University

**Neil Wenger, MD, MPH**

Division of General Internal Medicine/Health Services

Research, Department of Medicine

University of California, Los Angeles

**Consultant Jack Westfall, MD, MPH**

DARTNet Institute

**Statistician Chi-hong Tseng, PhD**

Division of General Internal Medicine/Health Services

Research, Department of Medicine

University of California, Los Angeles

**Analyst Gabriela Gaona, MPH**

DARTNet Institute

**Director of Programs Daphne York**

DARTNet Institute

**Postdoctoral Research Luis Manuel Olguin Valencia, PhD**

**Fellow** Department of Family Medicine

University of California, Los Angeles

**Project Coordinator Danielle Schramm, MSPH**

Department of Family Medicine

University of California, Los Angeles

## 3 INTRODUCTION

### 3.1 Study Rationale

Older adults vastly underutilize evidence-based preventive health services that are proven to reduce serious illness, morbidity and mortality. In fact, fewer than half of adults aged 65 and older are up-to-date on evidence-based cancer screenings and vaccinations recommended by expert committees (e.g., the USPSTF and CDC/ACIP). Those at greatest risk for receiving poor preventive care include racial and ethnic minority groups and persons of low socioeconomic status. Yet interventions to remedy this underutilization in older adults have mostly targeted individual preventive health services, rather than the totality of services needed by patients.

A great, yet underused opportunity exists to address the NIA’s calls for multilevel interventions to improve uptake of evidence-based screening services (NOT-OD-22-106 on “Increasing Uptake of Evidence-Based Screening in Diverse Adult Populations across the Lifespan”). This opportunity is amplified by Medicare’s establishment of the **Annual Wellness Visit** (AWV), a free-to-the-patient benefit that gives clinicians dedicated time to focus on evidence-based preventive care and to perform health risk assessments that may exceed the scope of other preventive visits. Despite its promise, less than one-fourth of eligible fee-for-service Medicare patients completed an AWV in 2017[^1^](#_ENREF_1) due to challenges at patient, clinician, and practice levels.[^2-11^](#_ENREF_2) Further, racial/ethnic disparities exist, with lower AWV utilization among racial/ethnic minority patients.[^12^](#_ENREF_12)

We developed a novel multi-level intervention to address the complexities of increasing AWV uptake at multiple levels: patient (demand), clinician (supply of services), office/nursing staff, and organization. Implemented at the practice level, the ‘Practice-Tailored AWV Intervention’ (PT-AWV) couples electronic health record (EHR) tools with tailored practice redesign tools and approaches, which include materials targeted for racial/ethnic minority patients. Our intervention creates practice changes that should persist over time, but data are lacking on the effect of AWVs on evidence-based interventions shown to affect health, on how long these changes persist, and on how the intervention can best be tailored for different types of practice settings.

### 3.2 Background

**Evidence-based preventive health services improve the health of populations.** Evidence-based preventive health care for older adults includes cancer screening, vaccinations, and counseling. Cancer is the second-leading cause of death globally and in the United States.[^13^](#_ENREF_13)^,^[^14^](#_ENREF_14) Screening tests for colorectal cancer,[^15-28^](#_ENREF_15) breast cancer, [^28-37^](#_ENREF_28) lung cancer,[^38-44^](#_ENREF_38) and abdominal aortic aneurysms[^28^](#_ENREF_28)^,^[^45-48^](#_ENREF_45) are proven to reduce mortality. Similarly, vaccinations to prevent pneumonia (pneumococcal vaccine),[^49-51^](#_ENREF_49) shingles (herpes zoster vaccine),[^52-54^](#_ENREF_52) flu (influenza vaccine)[^55-59^](#_ENREF_55) and tetanus[^60^](#_ENREF_60)^,^[^61^](#_ENREF_61) reduce illness-associated morbidity and mortality, particularly in older adults who are at increased risk of adverse outcomes from these illnesses.[^60-65^](#_ENREF_60) Counseling about topics such as advance care planning[^66^](#_ENREF_66)^,^[^67^](#_ENREF_67) decreases life-sustaining treatment and hospitalizations and increases goal-concordant patient care.[^68^](#_ENREF_68)^,^[^69^](#_ENREF_69)

**Older Americans underutilize preventive health services, with racial / ethnic minorities and low-income Americans at highest risk for poor preventive health care.** Fewer than half of adults aged 65 and older are up-to-date on a core set of preventive health services (flu vaccine in the past year, pneumonia vaccine (ever), colorectal screening, and mammogram in the past 2 years (for women)).[^70^](#_ENREF_70)^,^[^71^](#_ENREF_71) Racial and ethnic disparities are common. All racial / ethnic minorities have lower rates of pneumococcal, herpes zoster, and tetanus vaccinations than Whites.[^72^](#_ENREF_72)^,^[^73^](#_ENREF_73) Colorectal cancer incidence and mortality rates are highest in Blacks,[^74-77^](#_ENREF_74) but along with Hispanics and Asians, they are screened less frequently than Whites.[^78-82^](#_ENREF_78) Blacks also complete fewer mammograms than Whites.[^78^](#_ENREF_78) Adults with lower income and less education receive less preventive cancer screening,[^83^](#_ENREF_83)^,^[^84^](#_ENREF_84) and preventive health care quality and disparities vary widely by state and region.[^85^](#_ENREF_85)

**The Annual Wellness Visit gives clinicians dedicated time to focus on preventive health and is associated with greater use of preventive health services.** Medicare reimburses two different free-to-patient preventive health visits: 1) the Initial Preventive Physical Examination (IPPE), known as the “Welcome to Medicare Preventive Visit” (a one-time benefit);[^86^](#_ENREF_86) and 2) the Annual Wellness Visit (AWV) (a yearly benefit for patients after their first 12 months of Medicare Part B eligibility).[^87^](#_ENREF_87) This proposal refers to these visits collectively as AWVs, though the requisite tasks for the visits differ slightly.[^86^](#_ENREF_86)^,^[^87^](#_ENREF_87) Advance care planning discussions incur no patient cost-sharing when furnished during the AWV.[^88^](#_ENREF_88)^,^[^89^](#_ENREF_89) AWVs may be performed by many types of clinicians, including physicians, physician assistants, nurse practitioners, clinical nurse specialists, clinical pharmacists, health educators, registered dieticians, and nutrition professionals.[^90^](#_ENREF_90)

Patients with an AWV are more likely than those without an AWV to complete preventive health services such as: cancer screenings (e.g., colorectal, breast and cervical); vaccinations (e.g., influenza, pneumococcal, and herpes zoster); screening about tobacco use, fall risks, and sexually transmitted diseases; advance care planning; and abdominal aortic aneurysm screening.[^91-100^](#_ENREF_91) Interventions utilizing nurses, pharmacists, or team-based care to provide AWVs have increased rates of colorectal cancer screening, mammograms, pneumococcal vaccination, and bone density tests.[^97^](#_ENREF_97) However, it is unclear if observed associations between AWVs and increased preventive services use resulted from pre-existing trends toward increased utilization.[^101^](#_ENREF_101)

**AWVs are underutilized.** Despite its promise for increasing preventive health services, uptake of AWVs has been low, going from 7.8% of beneficiaries in 2011 to 24% in 2017.[^102-108^](#_ENREF_102) In 2015 over half of practices provided no AWVs, and only 23.1% provided them to at least 25% of their patients.[^109^](#_ENREF_109) Practices caring for underserved populations had the lowest adoption rates.[^109^](#_ENREF_109) Those least likely to have AWVs were Black or Hispanic, female, older, not married, living in rural areas, and had lower incomes, less education, and more chronic conditions.[^12^](#_ENREF_12)^,^[^98^](#_ENREF_98)^,^[^107-110^](#_ENREF_107)

**Barriers to preventive health services require solutions addressing both the supply and demand for the services.** Both patient and clinician barriers to preventive health use exist.[^2^](#_ENREF_2) ***Challenges to ‘demand’*** include poor patient awareness of the need for preventive health care or its coverage[^3-7^](#_ENREF_3) and insufficient clinician counseling.[^3^](#_ENREF_3) Many people, particularly those foreign-born, believe that cancer screening is needed only when they develop symptoms.[^111^](#_ENREF_111)^,^[^112^](#_ENREF_112) Some patients may not believe in the efficacy of preventive care services[^113^](#_ENREF_113)^,^[^114^](#_ENREF_114) or may fear having a test.[^115^](#_ENREF_115)^,^[^116^](#_ENREF_116) Others have low health literacy,[^117-119^](#_ENREF_117) language barriers,[^120^](#_ENREF_120)^,^[^121^](#_ENREF_121) transportation difficulty,[^11^](#_ENREF_11)^,^[^119^](#_ENREF_119) or live in a rural area with limited access to care.[^122-124^](#_ENREF_122) A major barrier is lack of a clinician’s recommendation, particularly among low-income populations and racial/ethnic minorities.[^3-5^](#_ENREF_3)^,^[^115^](#_ENREF_115)^,^[^119^](#_ENREF_119)^,^[^125-128^](#_ENREF_125) ***Challenges to the ‘supply’*** of preventive screenings include low clinician prioritization and poor knowledge about age-based screening recommendations.[^8-11^](#_ENREF_8) Clinician obstacles include a lack of systems to identify, track, and remind them about a patient’s need for preventive services.[^11^](#_ENREF_11) During time-limited office visits, acute or chronic conditions often supersede preventive health discussions.[^11^](#_ENREF_11)^,^[^71^](#_ENREF_71) Additional barriers include feeling overwhelmed by and having poor knowledge about complex AWV documentation and billing requirements.[^5^](#_ENREF_5)^,^[^6^](#_ENREF_6)

**Existing interventions to promote preventive health services and AWVs mostly fail to address the supply and demand for services.** The majority of published interventions enlisted a non-physician healthcare clinician to conduct AWVs, sometimes working in a team with physicians.[^5^](#_ENREF_5)^,^[^96^](#_ENREF_96)^,^[^97^](#_ENREF_97)^,^[^125^](#_ENREF_125)^,^[^129-133^](#_ENREF_129) These interventions may be difficult for under-resourced solo- to mid-size practices. They also do not address typical clinician barriers to ordering preventive health services (e.g., utilization of reminder or tracking systems) or patient barriers to utilizing the services. Multicomponent interventions have been shown to be more effective than single component ones for increasing preventive health services use and promoting patient adherence.[^134-139^](#_ENREF_134) They are recommended by the Community Preventive Services Task Force for increasing cancer screening,[^139^](#_ENREF_139) and are likely needed to improve overall use of preventive health services.

**Intervention sustainability is often not addressed.** The continued use of intervention components beyond an initial funding period is often not addressed due to limited study duration and funding, making it difficult to assess deterioration of sustainability over time.[^140-143^](#_ENREF_140) The long-term sustainability of interventions has been mixed though,[^144^](#_ENREF_144)^,^[^145^](#_ENREF_145) making intervention sustainability an important assessment to conduct for all interventions.

### 3.3 Risk/Benefit Assessment

#### 3.3.1 Known Potential Risks

The risks of all study procedures are minimal because the study involves no experimental medications or procedures. We will use data that are routinely collected as parts of clinical care for measuring the quality of clinical care.

The group receiving the intervention consists of practice clinicians, staff and administrators. This study poses no additional risks to these participants, and instead presents opportunities for improving the clinical care they provide. All recommended visits and patient assessments will be completed at the discretion of participating clinicians.

For the **population cohort** of patients, this study poses few risks. Patients may experience discomfort from engaging in Annual Wellness Visits and in recommended preventive health services, which are promoted by our intervention. This is unlikely to be serious because the intervention seeks to promote care that patients should already be receiving (but may not be), and patients can choose to not participate or to not complete recommended health services.

The primary risk for the population cohort is due to a potential data breach. With the combination of data safeguards, encryption, and transfer of limited data used in the study, this is highly unlikely (see **Section** [**11.8**](#_Data_Handling_and) on Data Handling and Record Keeping). Efforts to reduce risks associated with data transfer include adopting data minimization efforts and extracting only the data necessary for the project, strict adherence to regulations outlined in HIPAA, and regular monitoring of data security.

For patients, clinicians and staff participating in **semi-structured interviews** and **surveys**, the principal risks and associated strategies for protections against or minimizing these risks are:

1. Discomfort with answering semi-structured interview or survey questions, which may be seen as an intrusion of privacy or which may feel burdensome.

- The voluntary nature of participation will be emphasized to participants, and they will be told that they can skip questions if they feel uncomfortable or discontinue participation at any time.

1. Discomfort with audio recording of semi-structured interviews.

- Prior to starting each interview, we will emphasize that participation is voluntary. Participants will be told that they can withdraw from the study at any time, and that they can decline to answer questions if they feel uncomfortable. Audio recordings can be partially or completed erased if desired. At the end of the study, audio recordings will be either destroyed or modified to eliminate the possibility that study participants could be identified.

1. Breach of privacy.

- Standard measures will be taken to secure audio recordings of semi-structured interviews and survey data. This will include transfer of audio recordings to a UCLA secure network server within 24 hours and de-identification of verbatim transcriptions of the audio recordings. Lists of potential patient participants for recruitment will be maintained on secure network servers or a HIPAA-compliant data storage solution such as UCLA Health BOX. Survey data will be collected using UCLA Health approved survey platforms such as Qualtrics or REDCap.

All risks are unlikely to occur and are unlikely to be serious. The investigators have completed data collection methods involving similar protocols for multiple studies, including a pilot-study utilizing similar procedures.

*Alternative procedures.* The alternative to participating in the PT-AWV intervention is for practices to decline participation. The alternative to participating in semi-structured interviews and surveys is to not participate. Semi-structured interview participants may withdraw from participation at any time.

#### 3.3.2 Known Potential Benefits

Preventive health services are proven to improve population and individual health and are vastly underutilized by patients. If successful, this study will increase the use of Medicare AWVs in Medicare patients and decrease racial/ethnic disparities in use. We anticipate that increases in AWVs will result in increased patient use of preventive health services, and consequently improve population health and reduce mortality. Practices will benefit from the research due to increased revenue from providing Medicare AWVs and increased satisfaction from providing patients with better care.

#### 3.3.3 Assessment of Potential Risks and Benefits

The study poses few risks outside of what patients would experience during normal office visits. The minimal risks are outweighed by the benefits, which include improved population health and reduced mortality.

## 4 OBJECTIVES AND ENDPOINTS

| OBJECTIVES | ENDPOINTS | JUSTIFICATION FOR ENDPOINTS |
| --- | --- | --- |
| Primary |  |  |
| Evaluate the effect of ***the intervention*** (PT-AWV intervention) on:   1. AWV utilization 2. USPSTF and CDC/ACIP-recommended preventive services | **Primary endpoints:**  **AWV utilization** will be assessed by examining: the % of eligible patients completing an AWV (CPT codes G0438, G0439, G0468) or Initial Preventive Physical Examination (IPPE) (CPT code G0422) in the past 12 months.  **Completion of recommended preventive health services** will be measured using a composite preventive health services score (% of total recommended preventive health services that are up to date (of a maximum of 12 recommended services per patient)); measured on the patient-level  **Secondary endpoint:**  **Utilization of individual preventive health services**: % of patients up to date for each preventive health service listed in **Table 2** below. | These endpoints are the primary endpoints of interest.  AWVs mediate fulfillment of clinical endpoints on preventive health services.  Completion of recommended preventive health services can improve population health and reduce mortality. |
| Secondary |  |  |
| Evaluate the effect of ***the intervention*** (PT-AWV intervention) on reducing racial/ethnic disparities in AWV utilization. | As for the primary objective, **AWV utilization** will be assessed by examining: the % of eligible patients completing an AWV (CPT codes G0438, G0439, G0468) or Initial Preventive Physical Examination (IPPE) (CPT code G0422) in the past 12 months. | Secondary endpoints will demonstrate additional effects of the intervention and provide additional information to support the importance of the primary endpoints of AWV and preventive health service utilization |
| Tertiary/Exploratory |  |  |
| Tertiary: Evaluate factors affecting implementation and sustainability of the PT-AWV intervention tools and approaches, implementation strategies, and intervention effect in diverse practice settings. | Facilitators and barriers to intervention adoption and implementation (from semi-structured interviews [with patients, clinicians and staff] and clinician surveys) | These endpoints may suggest further hypotheses for later research and will provide information about causal mechanisms regarding uptake of AWVs |

| **Table 2. Preventive health services, recommended frequency and age, and evidence for use/no use*** | | | |
| --- | --- | --- | --- |
| **Outcome measure** | **Recommended frequency** | **Recommended Age** | **Grade^±^** |
| Vaccinations[^52^](#_ENREF_52) |  |  |  |
| Influenza[^146^](#_ENREF_146) | Yearly | 6 months of age or older |  |
| Herpes zoster (shingles)[^147^](#_ENREF_147) | Once (2 doses) | 50 or older |  |
| Pneumococcal (PCV20; PPSV23)[^148^](#_ENREF_148) | Once | 65 and older regardless of previous vaccination history |  |
| Tetanus (Td or Tdap)[^149^](#_ENREF_149) | Booster every 10 years | 19 and older |  |
| Cancer screening |  |  |  |
| Colorectal cancer | Every 1-10 years depending on type of screening | 45-49 years  50-75  76-85 | B  A  C |
| Mammogram | Every 2 years | Women 50 to 74 years  75 or older | B  I |
| Pap smear | Every 3-5 years | Women 21-65 years  Older than 65 | A  D |
| PSA (prostate) | Individualized decision (55-69)  Not recommended if >=70 | Men aged 55-69  Men 70 and older | C  D |
| Other screening |  |  |  |
| Ultrasound for abdominal aortic  aneurysm[^28^](#_ENREF_28)^,^[^45^](#_ENREF_45)^,^[^46^](#_ENREF_46) | Once | Men ages 65-75 who have ever smoked | B |
| Bone densitometry | At least once | Women aged 65 and older | B |
| Hepatitis C[^150^](#_ENREF_150) | Once | 18-79 | B |
| Alcohol misuse | Yearly | 18 or older | B |
| Tobacco use | Yearly | All adults | A |
| Depression[^151^](#_ENREF_151) | Yearly | All adults | B |
| Advance care planning[^66^](#_ENREF_66)^,^[^67^](#_ENREF_67) |  |  |  |
| ***** Based on USPSTF, CDC/ACIP guidelines,[^152-154^](#_ENREF_152) will adjust as needed to match guidelines at the time of data collection  ± A=recommended (high certainty of substantial net benefit); B=recommended (high certainty of moderate net benefit or moderate certainty of moderate to substantial net benefit); C=selectively offer based on professional judgement and patient preferences (moderate certainty of small net benefit); D=recommends against service (no net benefit or harms outweigh the benefits); I=current evidence is insufficient to assess balance of benefits and harms | | | |

## 5 STUDY DESIGN

### 5.1 Overall Design

This is a multi-site, stepped wedge cluster randomized controlled trial to evaluate the effect of the PT-AWV intervention on AWV and preventive health services use. The study will take place in 24 primary care practices around the United States (8 community-based, 8 academic, and 8 safety net practices). Recruitment and intervention implementation occur at the practice level. We hypothesize that the intervention will increase uptake of AWVs and use of preventive health services.

The **stepped wedge cluster design** is a pragmatic study design that includes an initial control period in which no clusters receive the intervention. Randomized clusters cross to the intervention condition at regular intervals, and there is a period at the end of the study where all clusters have been exposed to the intervention. Data are collected throughout the study so that each cluster contributes to both the control and intervention periods.[^155-157^](#_ENREF_155) The intervention will be implemented sequentially over 15 months, with 4 clusters of 6 practices each switching from control to intervention every 3 months. For each practice, intervention implementation will occur over a 6-month period. All practices will complete intervention implementation 15 months after the onset of intervention implementation in the first set of practices.

Outcomes for the primary and secondary endpoints will be assessed through EHR data extractions. The DARTNet Institute (co-investigator Wilson Pace, MD, PhD) will serve as the data coordinating center for the study. Co-investigator Elizabeth Callen, PhD at the American Academy of Family Physicians National Research Network (AAFP NRN) will clean, de-identify and create flags to develop analytic datasets. The AAFP NRN has established contracts and agreements with the DARTNet Institute that enable Dr. Callen to access DARTNet’s data. Dr. Callen will deliver limited datasets to investigators at the University of California, Los Angeles for analyses.

In addition to the procedures described above, we will evaluate implementation and sustainability of the evaluation (tertiary endpoints) by collecting qualitative semi-structured interview data on up to 10 patients per practice after an AWV, and up to 10 clinicians/staff per practice. Clinician and staff interviews will occur immediately, 12- and 24-months post-implementation. Clinicians also will be surveyed at those time points regarding their use of the intervention components (which ones, how often), perceived utility of the components, and barriers and facilitators to intervention implementation.

### 5.2 Scientific Rationale for Study Design

The stepped wedge cluster design is particularly suited to this study because: randomization is at the level of the practice (cluster) rather than at the level of the patient; the design will allow all participating practices to receive the intervention; and it would not be possible to implement the intervention simultaneously at all practices. [^155-157^](#_ENREF_155) Replication of the clinical trial will allow us to evaluate and compare intervention implementation and effects in different types of practice settings.

### 5.3 Justification for Intervention

**Intervention Pilot-Testing.** The investigators successfully pilot-tested the PT-AWV intervention in an organization consisting of 3 practice sites.[^158^](#_ENREF_158) All practices used the eMDs EHR platform. As shown in **Figure 2**, AWV utilization in the past 12 months (across all sites) went from 7% at baseline to 54% eight months post-intervention implementation. Two larger practices each increased AWVs by about 40%, while the smallest practice doubled its AWVs (from 15% to 30%) before dropping to 21%.

**Figure 2.**

**Effect of Practice-Tailored AWV Intervention on rates of AWVs, overall and by practice site**


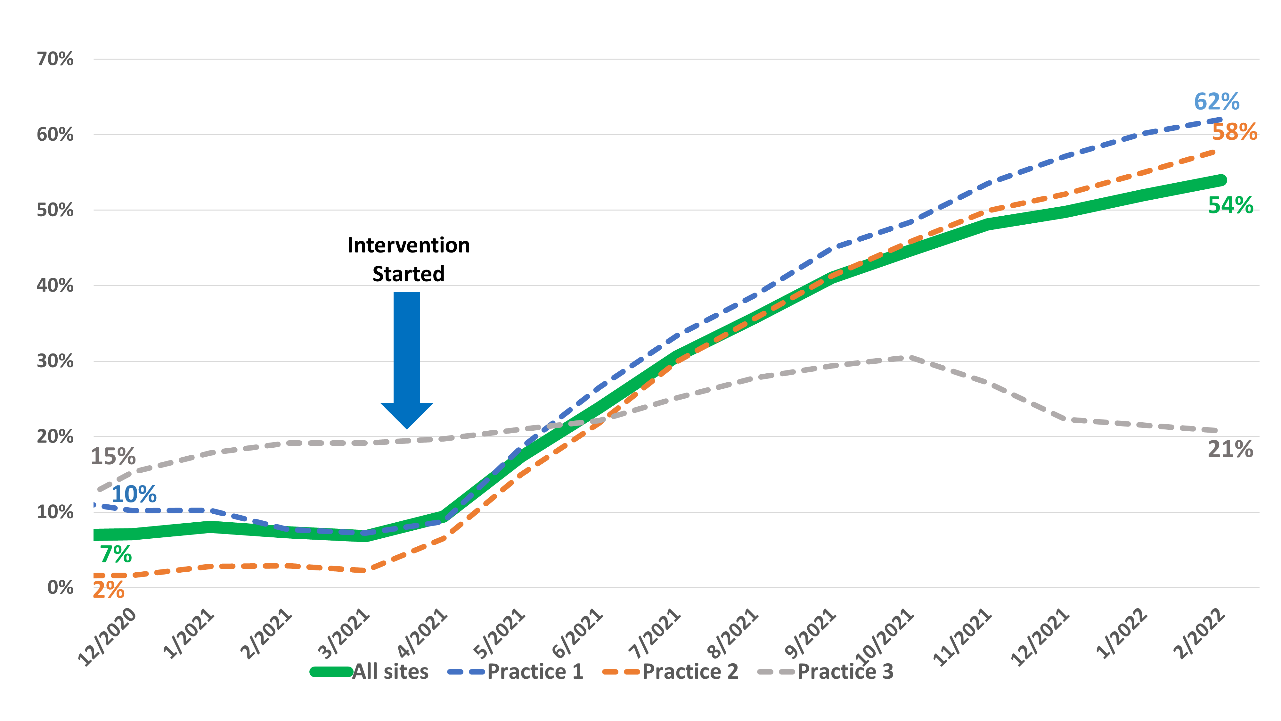


We have found that it is most effective to tailor the intervention to the needs of individual sites, and to allow sites to select the tools they wish to use. The minimum acceptable participation/exposure for intervention implementation is to work with the investigators to develop a practice-specific workflow for performing AWVs and to participate in clinician training sessions on AWV requirements and documentation.

### 5.4 End-Of-Study Definition

Practices reach the end of the study once they complete intervention implementation, contribute the requisite interviews with their patients, clinicians/staff, and contribute final EHR data extractions.

The end of the study is defined as 24 months after intervention implementation has been completed in all participating practices, as shown in the study Schema, [**Section 1.2**](#_1.2_Schema)**.**

## 6 STUDY POPULATION

### 6.1 Inclusion Criteria

***Practices*** must meet the following criteria to be included in the study:

1. Primary care practice
2. Provides care for Medicare patients

***Patients*** must meet all of the following criteria to be included in assessment of outcomes and semi-structured interviews:

1. Have Medicare insurance (and are therefore eligible for an AWV or Initial Preventive Physical Examination (IPPE))
2. Have had at least 1 encounter in the past 12 months at a participating practice

***Clinicians/Staff*** must meet the following criteria to be included in semi-structured interviews:

1. Work at a participating practice
2. Conduct, participate in, schedule, follow up on or supervise procedures for AWVs

Surveys will be given only to clinicians who work at a participating practice and conduct AWVs. Clinicians not currently conducting AWVs will be surveyed if their practice would like them to conduct AWVs.

### 6.2 Exclusion Criteria

Any patient who is deceased will be excluded from assessment of outcomes. For semi-structured interviews, participants will be excluded if they are unable to hear well enough to participate in a telephone interview or if the patient / family member reports that they are too sick.

### 6.3 Inclusion of Vulnerable Participants

#### 6.3.1 Participation of NIH Staff or family members of study team members

Not applicable

### 6.4 Inclusion of Pregnant Women, fetuses or neonates

Not applicable

### 6.5 Lifestyle Considerations

Not applicable

### 6.6 Screen Failures

Screen failures are defined as participants who are interested in participating in semi-structured interviews for this study but do not subsequently enter the study because they do not meet inclusion criteria. These individuals will not be rescreened.

### 6.7 Strategies for Recruitment and Retention

Healthcare organizations/practices will be recruited for the study. For the population cohort, there will be no direct patient recruitment. Outcomes will be extracted from EHRs and will not require active practice or patient participation. Patients will be recruited for one-time, semi-structured interviews. Clinicians and staff will be recruited for three sets of semi-structured interviews and clinicians for three sets of surveys. The interviews and surveys are not longitudinal and different clinicians/staff can participate at different time points. Retention strategies are not needed for this study.

Primary care practices. We have already obtained agreement for participation from 3 large organizations, each comprising at least 30-40 practice sites, and will select up to 8 from each organization to take part in this study.

Patients, clinicians, and staff will be recruited for semi-structured interviews. Clinicians will also be recruited for brief surveys. Informed consent for interviews and surveys will be provided by giving participants a study information sheet prior to the interview and/or obtaining oral consent.

*Patients* may be recruited in several ways:

- Identification of patients completing AWVs through EHR data extractions. The participating practice and/or a proxy designated by the practice (the DARTNet Institute or UCLA) will email or mail potential eligible patients a one-time recruitment letter and information sheet describing the study. Patients will be given the opportunity to phone or email the study team to opt-out of the study. A minimum of 1 week after the letter is sent, a study team member will telephone patients who do not opt-out for follow-up. Patients will receive up to 7 follow-up calls and/or emails, at least 1 week apart, up to 16 weeks after the letters are sent. Inquiries will be handled by the study team.
- Office staff and/or clinicians will invite patients to participate after they complete an AWV, by handing them an information flyer describing the interviews. The flyer will be used as the recruitment script.
- Office staff and/or clinicians will identify patients who previously completed an AWV and will invite them to participate in the study.

*Clinicians / staff*

Practice CEOs/administration/clinicians will have already agreed for their practice to participate in the study. For clinician/staff interviews, the practice lead will identify relevant parties participating in the process of scheduling, conducting, and following up on AWVs, and will ask them to speak to the study team for the interviews. The practice lead will assist with connecting relevant parties with the study team via email and/or will ask individuals to sign up for open interview slots. Clinicians will receive survey invitations from the study team and/or from their practice lead, practice administrator, or designated practice contact.

#### 6.7.1 Costs

Subjects will not incur any costs for their study participation.

#### 6.7.2 Compensation

Each practice will receive $2000 for their participation in the study, to be provided once they complete intervention implementation and have contributed the requisite number of semi-structured interviews and surveys for the study. This is meant to defray the cost of clinician and staff time for participating in assessments and trainings, and for the practice champion’s time to coordinate with the research team.

Subjects will receive $25 for participating in semi-structured interviews. Clinicians will not be compensated separately for surveys.

## 7 STUDY INTERVENTION(S) OR EXPERIMENTAL MANIPULATION(S)

### 7.1 Study Interventions(s) or Experimental Manipulations(s) Administration

#### 7.1.1 Study Intervention or Experimental Manipulation Description

***Conceptual Framework.*** The practice redesign tools and approaches of the PT-AWV intervention are based on principles consistent with the Practice Change and Development Model,[^159^](#_ENREF_159)^,^[^160^](#_ENREF_160) which attempts to transform culture and thinking to yield sustained changes in practice. The model has 4 major interdependent elements. Initial assessments with practices will yield information about the different elements of the model so that intervention strategies can be customized to practices’ unique characteristics, including staff workflow, resources, and EHR capability. The intervention has two major components. It combines the use of electronic health record (EHR) generated tools with practice redesign tools and approaches (**Table 3)**.

***EHR-based tools.*** EHR-based tools will be provided to practices (or “turned on”) at the beginning of the intervention implementation period and will be available for the duration of the study. UCLA and the DARTNet Institute will work with organizations and practices to modify and/or provide EHR-based tools, based on organizational/practice needs. The UCLA investigative team will work with UCLA population health team to make modifications in UCLA Health’s Epic EHR to provide capabilities below selected by the health system.

The EHR-based tools draw from EHR data to generate a patient registry that describes fulfillment of AWVs and recommended preventive health services. Practices can use registries for patient outreach at the point-of-care, or for prompting patients to schedule appointments for AWVs through contact by telephone, letters, text messages or patient portals. Tools that draw on the registries have features that can be customized based on practice staffing and preferred workflows. These include:

- Point-of-care reports to office staff, clinicians, and patients detailing services (e.g., AWVs, preventive health services) that are at goal, that are not at goal, or not performed.
- Tracking of patient completion of recommended AWVs and preventive health services, for example through a web-based dashboard.
- Patient notifications about the need for an AWV and/or preventive health services. Notifications and information tailored to patient race/ethnicity can be provided at the point-of-care as a personalized prevention plan (one of the requirements of an AWV), and through a letter or phone, email, or text-messaged reminders (to patients who agree to receive messages from the practice).
- Reminders for patients who do not complete clinician-ordered preventive services within a specified timeframe of the order (adjusted for local scheduling limitations) that can be sent via phone, email, or text messaging.

***Practice redesign tools and approaches*** involve designing workflows to help practices use EHR tools and training members in the practice on using the workflows and on completing assigned tasks when patients arrive for an AWV. Training sessions will be performed via a video-conferencing platform. Resources include templates for required AWV components such health risk assessments and patient personalized prevention plans, patient messaging about AWVs, clinician documentation templates, and patient-targeted educational materials about the need for different preventive health services. There will be a focus on materials tailored to racial/ethnic minority patients and in non-English languages (drawn from existing materials found on the internet from expert organizations and the scientific literature). Resources also include sample workflows, including those developed by other practices. There also may be an option to notify patients who fail to complete recommended services, with the method of communication tailored to patient preferences. Feedback reports on rates of completion of AWVs and preventive health services, coupled with recommendations for continued improvements, will be provided monthly or quarterly (based on practice preference).

With an eye toward future broad dissemination of the intervention, we will modify our original intervention by posting all resources on a **study website** so that participating practices and patients can browse, select and download relevant templates and materials. Video recordings of clinician and staff training sessions will be available. As we learn more about what works in different practice settings and how people orient to the website, we will refine and update available materials, add to sample practice workflows, and modify instructions for using the website. Our larger goal is for the website to stand alone as a starting point for practices to access on their own, without requiring guidance from outside investigators.

**Tailoring of PT-AWV intervention components for practices**. Implementation of the PT-AWV intervention (the intervention) will take place at the practice level over 6 months.

The exact content of the intervention will be tailored based on each practice’s individual needs. For example, one practice may ask staff to use the AWV patient registry to invite patients to schedule a future AWV, while another practice may use the registry at the point-of-care to add an AWV to a visit scheduled on that day (if a clinician has availability). Similarly, practices will customize their selection of practice redesign tools and approaches and will work with the investigative team to tailor their workflows to match their individual resources and capabilities. **Table 3** describes examples of options for intervention tailoring.

| **Table 3. Major Intervention Tools and Approaches and Options for Tailoring *** | |
| --- | --- |
| **Tools / Approaches to address identified barriers to AWV use** | **Examples of Options for Tailoring** |
| **EHR-based tools** (implementation of tools depends on EHR capabilities and potential for modification) | |
| Patient registry to identify those without an AWV in the past 12 months and that includes information on preventive health services use | - Registry can be embedded in EHR or presented as a separate electronic “dashboard” - Practices may choose to receive EHR notifications |
| Point-of-care information regarding AWV and preventive service completion | - Practices select to receive point-of-care information electronically (e.g., in EHR, through dashboard) or on paper |
| Individualized patient preventive health plans | - Practices can select delivery format (paper, electronic) of preventive health plans |
| Message patients about need for services | - Practices can select timing and delivery method (EHR patient portal, postal mail, text message) of patient messages and reminders |
| Patient reminders if ordered services not completed |  |
| **Practice redesign tools and approaches** | |
| **Workflows (developed iteratively based on practice preferences and feedback)** | |
| For using EHR-based patient registry | Practices develop desired workflows for using patient registry to invite and schedule patients for AWVs  - may invite patients for AWVs by phone, text message, or via patient EHR portal |
| For recommending / scheduling patients for AWVs when patients present in-person | Practices choose who should recommend / schedule patients for AWVs when they present for other types of office visits |
| For accommodating AWVs in clinician schedules | - Practices decide type of clinician(s) who will conduct AWVs (physician, physician assistant, nurse practitioner, other) - Practices determine whether they will add AWVs to existing follow-up visits for other issues - Practices decide whether AWVs will be conducted in-person versus by telehealth - Practices choose amount of time allocated for AWVs |
| For performing tasks associated with AWVs | - Practices choose how much time before AWV to ask patients to arrive - Practices define strategies to complete time-consuming required health risk assessment. Approaches include: - Send form to patients via email, postal mail, EHR portal - Staff to complete by phone - Complete in office (practices opt to complete on paper, iPad or phone, verbally; who should complete with patient) |
| For performing assessments such as for fall risk, dementia screening (instruments and trainings for select tasks are available) | - Practices can choose who will perform assessments, what instruments to use; whether to offer training for staff on selected instruments |
| **Templates for documentation, collection, and delivery of Medicare-required information for AWVs** (practices/clinicians may elect to use only selected elements of templates) | |
| Health risk assessment templates (includes question bank) | - Practices can choose which questions they want to ask - Can include assessment of social determinants of health |
| Documentation templates that include required components of AWVs | - Clinicians tailor content of their templates based on personal preferences |
| EHR data collection package (e.g., SmartSet for Epic EHR) | - Practices can modify content of EHR data collection package and order of components |
| Preventive health summary templates | - Practices may choose content and formatting of preventive health summaries from existing examples or modify based on practice/clinician/patient preferences |
| **Clinician and staff training sessions** | |
| Clinician and staff PowerPoint presentations (contain information on benefits of AWVs, terminology, billing strategies, visit requirements, presentation of practice-developed workflow and role-specific tasks) | - Practices select number of training sessions, duration, participants (e.g., clinician, staff, combined) - Training sessions can be group, individual, or both - Information presented tailored based on clinician / practice motivation to provide care |
| **Other tools** | |
| ***Clinician-targeted*** |  |
| Opportunity to use data to fulfill AAFP  Maintenance of Certification practice  improvement activity | - Clinicians can choose data/metric they want to use to fulfill practice improvement activity |
| FAQs about AWVs (for clinicians) | - Practices can select frequency and modes (e.g., email, text messaging) chosen for dissemination of written materials |
| Summary of elements of IPPE and AWVs |  |
| Clinician billing tips |  |
| ***Patient-targeted*** |  |
| Messaging for contacting / informing  patients about importance of AWVs (from  AAFP) | - Practices can choose whether / how / when to send patients information - Practices may choose to make patient-facing materials available in waiting room, have office staff or clinician hand to patient during visits, mail to those on registry - Information provided to patients about preventive health screenings can be tailored by patient race/ethnicity |
| FAQs about AWVs (for patients) |  |
| Educational materials on common cancer  screenings, vaccinations (pulled from  existing sources such as the CDC) |  |
| **Feedback reports on rates of AWV and preventive health services completion**** | |
| Practice-level reports | - Practices may elect to receive reports monthly or quarterly - Practices may choose different metrics to review |
| Clinician-level reports | - Reports may compare clinicians to other clinicians in their practice (either de-identified or identifiable), or to others in the study |
| * Practices choose which tools they want to use and how they want to tailor each selected tool; selection and tailoring is based on individual practice needs and existing resources; practices are not required to use any specific tools | |

#### 7.1.2 Administration

**Initial practice assessments*.*** Initial practice assessments will be conducted prior to intervention implementation. An ***on-site practice champion*** identified by the practice will work with the investigative team to champion the intervention and its continued implementation and use, and ensure completion of all study assessments. The practice champion may be a physician, pharmacist, office manager, nurse, or other personnel.

Practice assessments will typically involve 2-3 meetings of about 30-60 minutes each, during which the investigative team will gather information regarding the practice’s motivation, resources, facilitators to conducting AWVs, current practice workflows and templates for AWVs, preferred workflows, and opportunities for change. Assessments will be performed via Zoom video conferencing, which have proven effective and efficient for intervention implementation and data collection across multiple sites,[^161^](#_ENREF_161) and enhances the potential for future widespread intervention dissemination. The study team will use information from the assessments to develop a prototype workflow, which they will present to the practice for discussion and revision. The investigators will also assess practice interest in using the resources available for download on the study website, introduce the website, and provide practices with the materials desired, as well as the website link so that they can browse, download, and use other desired resources. Discussions also will focus on selecting and tailoring EHR-based tools for their practice.

**Intervention implementation*.*** Once initial practice assessments have been completed, the practices have developed a workflow for conducting AWVs, and have selected their desired tools, the intervention will be implemented over 6 months. Intervention implementation will consist of “turning on” selected EHR-based tools, providing access to selected documentation and other templates, and training practice members on the use of these tools. Practice training sessions will occur during this period on the established practice-specific AWV workflow, benefits of AWVs, documentation requirements, services that clinicians can perform with the AWV without incurring extra patient costs, and potential billing strategies. The research team will interface with the practice champion regularly (on average likely about once every 1-2 months based on our experiences with our pilot practices) during the 6-month implementation period to identify and remedy barriers to implementation.

The investigative team will collate information from all sites on strategies for increasing AWVs and overcoming barriers to conducting AWVs and will share pertinent insights in a quarterly newsletter for practices that have already started intervention implementation. Practices will receive feedback reports on their AWV and preventive health services completion rates starting about 4-6 months after they begin intervention implementation until 12 months post-implementation. Practices will determine the desired frequency of these reports, which can be provided monthly to quarterly.

### 7.2 Fidelity

#### 7.2.1 Interventionist Training and Tracking

Variability of intervention implementation is expected because the intervention will be tailored by site. We will track dates of completion of meetings and trainings.

### 7.3 Measures to Minimize Bias: Randomization and Blinding

The study statistician will use stratified randomization to assign the order and timing of intervention implementation in practices. Stratification will be by organization to ensure that each cluster contains two practices from each organization. Randomization within each strata will be by simple randomization.[^162^](#_ENREF_162) Randomization will be performed before any practices are engaged in intervention implementation activities. Blinding procedures are not applicable for this study.

### 7.4 Study Intervention/Experimental Manipulation Adherence

The study team will assess and track details of all interactions with practices, including attendance at training sessions, and practice reports of use of intervention materials.

To remain an active participant in the study, practice facilitators must engage with and meet with the study team throughout the intervention implementation period.

### 7.5 Concomitant Therapy

Not applicable

#### 7.5.1 Rescue Therapy

Not applicable

## 8 STUDY INTERVENTION/EXPERIMENTAL MANIPULATION DISCONTINUATION AND PARTICIPANT DISCONTINUATION/ WITHDRAWAL

### 8.1 Discontinuation of Study Intervention/Experimental Manipulation

These are not applicable because the trial has low or negligible risk of meeting the usual criteria for stopping a study for safety, early success or futility. However, the trial may be stopped if external circumstances (including but not limited to national or global events) make continuation impossible. Reasons for discontinuation would be communicated to the NIH.

### 8.2 Participant Discontinuation/Withdrawal from the Study

Practices are free to withdraw from participation in the study at any time upon request. Practices that withdraw from the study will be replaced.

### 8.3 Lost to Follow-up

Not applicable

## 9 STUDY ASSESSMENTS AND PROCEDURES

### 9.1 Screening Procedures

**9.1.1 Screening activities performed prior to obtaining informed consent**

Minimal risk activities that may be performed before a participant has provided oral informed consent or has been given an information sheet regarding semi-structured interviews include the following:

- EHR data extractions to identify a list of eligible participants.
- Email, written, in person or telephone communications with prospective subjects.

**9.1.2 Screening activities performed after a consent for screening has been obtained**

Not applicable

### 9.2 Study Evaluations & Procedures

*EHR data extractions to identify patients for interviews* will be performed to identify eligible patients for semi-structured interviews. Information obtained through the extractions will include patient names, contact information, and date of birth. Extractions will occur within about 1-6 months prior to patient participation in interviews. Clinicians/staff will be identified by practices for participation.

*Clinician/staff interviews* will be conducted via telephone or Zoom videoconferencing and will last approximately 30 minutes. Interviews will be audio-recorded and transcribed verbatim for analysis. Interviews are not longitudinal. Different clinicians/staff may participate at different time points. Interviews will be conducted by a team member with qualitative research expertise.

*Patient interviews* will be conducted via telephone and will last approximately 45-60 minutes. These one-time interviews will be audio-recorded and transcribed verbatim for analysis. Interviews will be conducted by a team member with qualitative research expertise.

*Clinician surveys* will be administered via REDCap or Qualtrics, and will take approximately 5-10 minutes or less to complete. Clinicians will be identified by practice leads for participation.

*Data extractions to assess primary and secondary outcomes*. The DARTNet Institute will serve as the data coordinating center for this study. Organizations will either send DARTNet limited datasets for standardization, or DARTNet will work with the institutions to extract the requisite data to assess study outcomes, which include completion of AWVs and the preventive health measures depicted in [**Section 4**](#_4_OBJECTIVES_AND) (**Table 2**). Dr. Callen (co-investigator) will process data and clean the data, help create analytic datasets, and will transfer limited datasets to UCLA for analysis. Data will be extracted at baseline and in July 2025, July 2026, and July 2027.

#### 9.2.1 Biospecimen Evaluations

Not applicable

#### 9.2.2 Samples for Genetic/Genomic Analysis

##### 9.2.2.1 Description of the scope of genetic/genomic analysis

Not applicable

##### 9.2.2.2 Description of how privacy and confidentiality of medical information/biological specimens will be maximized

Not applicable

##### 9.2.2.3 Management of Results

Not applicable

##### 9.2.2.4 Genetic counseling

Not applicable

### 9.3 Safety Assessments

Not applicable

### 9.4 Adverse Events and Serious Adverse Events

#### 9.4.1 Definition of Adverse Event

Adverse events are not anticipated because the intervention is performed at the level of the practice, and not with patients. The risks of the study are minimal as the intervention promotes practices that are part of routine clinical care.

Adverse event means any untoward medical occurrence associated with the use of an intervention in humans, whether or not considered intervention-related (21 CFR 312.32 (a)).

#### 9.4.2 Definition of Serious Adverse Events (SAE)

An adverse event (AE) or suspected adverse reaction is considered "serious" if, in the view of either the investigator or sponsor, it results in any of the following outcomes: death, a life-threatening adverse event, inpatient hospitalization or prolongation of existing hospitalization, a persistent or significant incapacity or substantial disruption of the ability to conduct normal life functions, or a congenital anomaly/birth defect. Important medical events that may not result in death, be life-threatening, or require hospitalization may be considered serious when, based upon appropriate medical judgment, they may jeopardize the participant and may require medical or surgical intervention to prevent one of the outcomes listed in this definition. Examples of such medical events include allergic bronchospasm requiring intensive treatment in an emergency room or at home, blood dyscrasias or convulsions that do not result in inpatient hospitalization, or the development of drug dependency or drug abuse.

#### 9.4.3 Classification of an Adverse Event

##### 9.4.3.1 Severity of Event

For adverse events (AEs) not included in the protocol defined grading system, the following guidelines will be used to describe severity.

- **Mild** – Events require minimal or no treatment and do not interfere with the participant’s daily activities.
- **Moderate** – Events result in a low level of inconvenience or concern with the therapeutic measures. Moderate events may cause some interference with functioning.
- **Severe** – Events interrupt a participant’s usual daily activity and may require systemic drug therapy or other treatment. Severe events are usually potentially life-threatening or incapacitating. Of note, the term “severe” does not necessarily equate to “serious”.

##### 9.4.3.2 Relationship to Study Intervention/Experimental Manipulation

All adverse events (AEs) will have their relationship to study procedures, including the intervention, assessed by an appropriately trained clinician based on temporal relationship and his/her clinical judgment. The degree of certainty about causality will be graded using the categories below.

**Related** – The AE is known to occur with the study procedures, there is a reasonable possibility that the study procedures caused the AE, or there is a temporal relationship between the study procedures and the event. Reasonable possibility means that there is evidence to suggest a causal relationship between the study procedures and the AE.

**Not Related** – There is not a reasonable possibility that the study procedures caused the event, there is no temporal relationship between the study procedures and event onset, or an alternate etiology has been established.

*OR*

**Definitely Related** – There is clear evidence to suggest a causal relationship, and other possible contributing factors can be ruled out. The clinical event, including an abnormal laboratory test result, occurs in a plausible time relationship to study procedures administration and cannot be explained by concurrent disease or other drugs or chemicals. The response to withdrawal of the study procedures should be clinically plausible. The event must be pharmacologically or phenomenologically definitive.

**Probably Related** – There is evidence to suggest a causal relationship, and the influence of other factors is unlikely. The clinical event, including an abnormal laboratory test result, occurs within a reasonable time after administration of the study procedures, is unlikely to be attributed to concurrent disease or other drugs or chemicals, and follows a clinically reasonable response on withdrawal.

**Potentially Related** – There is some evidence to suggest a causal relationship (e.g., the event occurred within a reasonable time after administration of study procedures). However, other factors may have contributed to the event (e.g., the participant’s clinical condition, other concomitant events). Although an AE may rate only as “possibly related” soon after discovery, it can be flagged as requiring more information and later be upgraded to “probably related” or “definitely related”, as appropriate.

**Unlikely to be related** – A clinical event, including an abnormal laboratory test result, whose temporal relationship to study procedures administration makes a causal relationship improbable (e.g., the event did not occur within a reasonable time after administration of the study procedures) and in which other drugs or chemicals or underlying disease provides plausible explanations (e.g., the participant’s clinical condition, other concomitant treatments).

**Not Related** – The AE is completely independent of study procedures administration, and/or evidence exists that the event is definitely related to another etiology. There must be an alternative, definitive etiology documented by the clinician.

##### 9.4.3.3 Expectedness

A clinician with appropriate expertise in primary care will be responsible for determining whether an adverse event (AE) is expected or unexpected. An AE will be considered unexpected if the nature, severity, or frequency of the event is not consistent with the risk information previously described for the study procedures.

#### 9.4.4 Time Period and Frequency for Event Assessment and Follow-Up

While unlikely, the occurrence of an adverse event (AE) or serious adverse event (SAE) may come to the attention of study personnel during interviews of a study participant.

All AEs, not otherwise precluded per the protocol, will be captured on the appropriate case report form (CRF). Information to be collected includes event description, time of onset, clinician’s assessment of severity, relationship to study procedures (assessed only by those with the training and authority to make a diagnosis), and time of resolution/stabilization of the event. All AEs occurring while on study will be documented appropriately regardless of relationship. All AEs will be followed to adequate resolution.

Any medical or psychiatric condition that is present at the time that the participant is screened will be considered as baseline and not reported as an AE. However, if the study participant’s condition deteriorates at any time during the study, it will be recorded as an AE.

Changes in the severity of an AE will be documented to allow an assessment of the duration of the event at each level of severity to be performed. Documentation of onset and duration of each episode will be maintained for AEs characterized as intermittent.

The Project Coordinator and/or research associate will record all reportable events that occur during the one-time study participation for semi-structured interviews. Events will be followed for outcome information until resolution or stabilization.

#### 9.4.5 Adverse Event Reporting

Unexpected adverse events, serious adverse events, and other reportable events will be reported to the UCLA Institutional Review Board by the PI or the Project Coordinator. Decisions regarding relatedness and severity will be discussed among the investigative team.

An unanticipated problem that resulted in a subject’s death or was potentially life-threatening will be reported to the IRB within a shorter time frame than other unanticipated problems that were not life-threatening.  We will follow UCLA IRB guidelines in order to satisfy the requirement for *prompt* reporting:

1. Unanticipated problems that are serious adverse events will be reported to the UCLA IRB within 1 week of the investigator becoming aware of the event.
2. Any other unanticipated problem will be reported to the IRB within 2 weeks of the investigator becoming aware of the problem.
3. All unanticipated problems will be reported to appropriate institutional officials (as required by an institution’s written reporting procedures), the supporting agency head (or designee), and the UCLA Office of Human Research Protection within one month of the IRB’s receipt of the report of the problem from the investigator.

#### 9.4.6 Serious Adverse Event Reporting

In consultation with the PI, a trained member of the study team will be responsible for conducting an evaluation of a serious adverse event and shall report the results of such evaluation to the UCLA Institutional Review Board (IRB).

#### 9.4.7 Events of Special Interest

Not applicable

#### 9.4.8 Reporting of Pregnancy

Not applicable

### 9.5 Unanticipated Problems

#### 9.5.1 Definition of Unanticipated Problems (UP)

Any incident, experience, or outcome that meets **all** of the following criteria:

- Unexpected in terms of nature, severity, or frequency given (a) the research procedures that are described in the protocol-related documents, such as the Institutional Review Board (IRB)-approved research protocol and informed consent document; and (b) the characteristics of the participant population being studied; and
- Related or possibly related to participation in the research (“possibly related” means there is a reasonable possibility that the incident, experience, or outcome may have been caused by the procedures involved in the research); and
- Suggests that the research places participants or others (which many include research staff, family members or other individuals not directly participating in the research) at a greater risk of harm (including physical, psychological, economic, or social harm) than was previously known or expected.

#### 9.5.2 Unanticipated Problem Reporting

The investigator will report unanticipated problems (UPs) to the UCLA Institutional Review Board (IRB) according to the UCLA IRB’s policies.

## 10 STATISTICAL CONSIDERATIONS

### 10.1 Statistical Hypothesis

- ***Primary Endpoints***:

We hypothesize that the intervention (PT-AWV intervention) will result in:

- 1. greater AWV utilization (% of eligible patients completing an AWV or IPPE in the past 12 months) at 12, 18 and 24 months after intervention implementation
  2. patients being up to date on a greater percentage of their total number of recommended services (of a maximum of 12 recommended services per patient) at 12, 18 and 24 months after intervention implementation;

We also hypothesize that the PT-AWV intervention will reduce racial/ethnic disparities in AWV utilization. Alternatively, our null hypotheses are that there will be no difference in AWV or USPSTF and CDC/ACIP-recommended service utilization with implementation of the PT-AWV intervention and that there will be racial/ethnic disparities in AWV utilization.

- ***Secondary Endpoint***(s):

We hypothesize that the PT-AWV intervention will result in a greater percentage of patients who are up to date on each of the individual preventive health services listed in [Section 4.0](#_4_OBJECTIVES_AND) **Table 2**. Alternatively, our null hypothesis is that there will be no difference in utilization in the USPSTF and CDC/ACIP-recommended services examined.

### 10.2 Sample Size Determination

In our pilot practices, the PT-AWV intervention increased AWVs from 8% at baseline to 54% eight months post-intervention in AWV-eligible patients (n=213, 576 and 639 patients at baseline in the 3 practices). Practices agreeing to participate in this project range in size from about 175-2700 eligible patients and have baseline AWV rates of about 16-45%, with most patients belonging to practices with rates under 35%. At the population level, we conservatively estimate our sample size to be a mean of about 1300 patients per practice. Behaviors of practices recruited from the same healthcare organizations may be correlated, so we will adjust power analyses using intra-class correlation (ICC). Based on our preliminary data, intra-class correlation for site was 0.03 and within subject correlation was -0.003 for AWV use one year apart. Since the stepped wedge design is relatively insensitive to ICC (because it is similar to a pre-test/post-test design), we conservatively estimate ICCs from .03 to .05 in our calculations and assume that ICC is mostly due to repeated patients seen in the same practice. Clinically meaningful increases would conservatively be 10% for all outcomes. The overall treatment effect is >99% for a 10% increase in AWVs and in the composite preventive health services score, based on a type-I error rate α = 0.05, 800-1300 patients per practice, and ICC=0.03 and 0.05 for 24 practices.

**Table 4** demonstrates power calculations to detect a meaningful treatment effect of 10% increase outcomes for a sample of 24 practices, with type-I error rate α = 0.05. We have >99% power to detect an overall improvement of 10% in AWV completion rates and the composite preventive health score in all scenarios from baseline to 12 months post-intervention.

| **Table 4. Power for treatment effect of 10% increase, type-I error rate α = 0.05, based on # monthly eligible patients and baseline rates of outcomes, and ICC for 24 practices** | | | |
| --- | --- | --- | --- |
| **Outcome / Mean # patients per site** | **Baseline rate, %** | **Power with ICC 0.05, %** | **Power with ICC 0.03, %** |
| ***AWV*** |  |  |  |
| 800 patients / practice | 10 | >99 | >99 |
|  | 30 | >99 | >99 |
|  | 50 | >99 | >99 |
| 1200 patients / practice | 10 | >99 | >99 |
|  | 30 | >99 | >99 |
|  | 50 | >99 | >99 |
| 1500 patients / practice | 10 | >99 | >99 |
|  | 30 | >99 | >99 |
|  | 50 | >99 | >99 |
| ***Composite preventive health score*** |  | >99 | >99 |
| 800 patients / practice |  | >99 | >99 |
| 1200 patients / practice |  | >99 | >99 |
| 1500 patients / practice |  | >99 | >99 |

Our pilot data suggested a decrease in ***racial/ethnic disparities*** in AWV utilization: at baseline, 7.7% of White patients and 4.9% of non-Whites completed AWVs (p=0.09), whereas 6 months post-implementation, 49.8% of White patients and 50.5% of non-Whites completed AWVs (p=0.63).

**Table 5** demonstrates power calculations to detect a 5% reduction in racial/ethnic disparities for AWVs with a sample of 24 practices, ICC=0.05 and type-I error rate α = 0.05. We will have >99% power to detect a 5% reduction in racial/ethnic disparities in AWVs between White and Hispanic patients and >99% power to detect a 5% reduction in disparities between White and Black patients for AWV completion. This calculation assumes the intervention effect is about 10% for White patients and 15% for Black and Hispanic patients, and that about 60%, 20% and 15% of the study population are White, Hispanic, and Black. For the composite preventive health services score power calculation, we further assume that on average each patient is eligible for > 8 preventive health services.

| **Table 5. Power for detecting 5% reduction in racial/ethnic disparities for AWVs and composite preventive health services score, with a type-I error rate α = 0.05 and ICC=0.05, based on patients/practice and baseline rates of outcomes** | | | | |
| --- | --- | --- | --- | --- |
| **Outcome** | **Baseline rate, %** | **Baseline rate, %** | **Power for Black-White disparity reduction, %** | **Power for Hispanic-White disparity reduction, %** |
| ***AWV*** | ***White*** | ***Black/Hispanic*** |  |  |
| 800 patients / practice | 30 | 10 | >99% | >99% |
|  |  | 15 | >99% | >99% |
|  |  | 20 | >99% | >99% |
|  | 40 | 10 | >99% | >99% |
|  |  | 15 | >99% | >99% |
|  |  | 20 | >99% | >99% |
|  | 50 | 10 | >99% | >99% |
|  |  | 15 | >99% | >99% |
|  |  | 20 | >99% | >99% |
| 1200 patients / practice | 30 | 10 | >99% | >99% |
|  |  | 15 | >99% | >99% |
|  |  | 20 | >99% | >99% |
|  | 40 | 10 | >99% | >99% |
|  |  | 15 | >99% | >99% |
|  |  | 20 | >99% | >99% |
|  | 50 | 10 | >99% | >99% |
|  |  | 15 | >99% | >99% |
|  |  | 20 | >99% | >99% |
| 1500 patients / practice | 30 | 10 | >99% | >99% |
|  |  | 15 | >99% | >99% |
|  |  | 20 | >99% | >99% |
|  | 40 | 10 | >99% | >99% |
|  |  | 15 | >99% | >99% |
|  |  | 20 | >99% | >99% |
|  | 50 | 10 | >99% | >99% |
|  |  | 15 | >99% | >99% |
|  |  | 20 | >99% | >99% |
|  |  |  |  |  |
| ***Composite preventive health services score*** |  |  |  |  |
| 800 patients / practice | 40 | 10 | >99% | >99% |
|  |  | 15 | >99% | >99% |
|  |  | 20 | >99% | >99% |
| 1200 patients / practice | 40 | 10 | >99% | >99% |
|  |  | 15 | >99% | >99% |
|  |  | 20 | >99% | >99% |
| 1500 patients / practice | 40 | 10 | >99% | >99% |
|  |  | 15 | >99% | >99% |
|  |  | 20 | >99% | >99% |

### 10.3 Populations for Analyses

All patients in each practice meeting inclusion criteria will be included in analyses, as practices (not patients) are the ones exposed to the intervention. The patient cohort to be analyzed for the primary endpoints (AWV utilization and composite preventive health services score) consists of patients:

- aged 50 and older
- have Medicare insurance
- have had at least 1 encounter in the past 12 months at a participating practice site
- are not deceased.

Multiple cohorts exist for our secondary endpoints, as these cohorts vary based on the preventive health service assessed. Cohorts for the secondary outcomes include those meeting the inclusion criteria for the primary outcomes who meet additional eligibility requirements for each preventive service listed in [Section 4.0](#_4_OBJECTIVES_AND) **Table 2**. We will use HEDIS and CMS metric specifications to specify inclusion and exclusion criteria for each preventive health service. As an example, the patient cohort for mammography includes the cohort for the primary outcomes who are female, aged 50 to 74, and do not meet HEDIS metric exclusion criteria.

### 10.4 Statistical Analyses

#### 10.4.1 General Approach

Descriptive statistics will be used to describe demographics of the primary patient cohort: age (means with standard deviations; range), gender (percentages), and race/ethnicity (percentages). For each practice and organization, we will summarize use of AWVs and preventive health services at baseline (Month 0) and every 6 months up to the end of the study (24 months after the completion of intervention implementation in the last set of practices). We will also use run charts to illustrate the monthly percentage of eligible patients who utilized AWVs and each preventive health service assessed ([Section 4.0](#_4_OBJECTIVES_AND) **Table 2**), as well as the composite preventive health services score.

**Linear mixed effects models** will be used to assess the effect of the intervention on the use of AWVs (primary endpoint), on the use of individual preventive health services (secondary endpoints), and on the composite preventive health services score (primary endpoint).

#### 10.4.2 Analysis of the Primary Endpoints

The primary endpoints are AWV utilization and the composite preventive health services score **(Table 6)**.

| **Table 6. Patient cohorts, primary endpoints, and timing of measurement** | | |
| --- | --- | --- |
| **Cohort** | **Endpoints** | **Time Frame** |
| Medicare patients aged 50+, with 1 or more visits in the past 12 months, not deceased | 1) ***AWV utilization*** (% of patients who completed an AWV or IPPE (CPT codes G0438, G0439, G0402)) within 12 months; 2) ***Composite preventive health services score*** (% of recommended services that are up to date) | Monthly |

***Primary objectives (examine effect of PT-AWV intervention on increasing AWVs and preventive health services utilization in diverse practice settings at 12, 18 and 24 months post-implementation)*:** we will use **linear mixed effects models** to assess the effect of the intervention on the use of AWVs and on the composite preventive health services score. These models account for correlation between repeated observations in the same cluster and the confounding effects of time and treatment. For example, with $Y_{ijt}$ as the indicator of AWV completion for patient i from cluster j at time t, the model is:

$Y_{ijt}=\mu+\alpha_{i}+\gamma_{j}{+ \beta}_{t}+X_{ijt}\theta+ Z_{ijt} + \varepsilon_{ijt}$,

where $\mu$ is the intercept,$\gamma_{i}$ and $\alpha_{j}$ are the random intercepts for subject i and cluster j with $\alpha_{j}\sim N\left( 0, \sigma_{\alpha}^{2} \right), \gamma_{j}\sim N\left( 0, \sigma_{\gamma}^{2} \right),$ $\beta_{t}$ is the fixed time effect, $X_{ijt}$ is the indicator of treatment mode for subject i cluster j at month t, $\theta$ represents the treatment effect, $Z_{ijt}$ is pre-specified patient and practice level covariate, and $\varepsilon_{ijt}$ is the residual with $\varepsilon_{ijt}\sim N\left( 0, \sigma_{\varepsilon}^{2} \right)$. We will approximate the secular time trend $\beta_{t}$ linearly in time with $\beta t$. Piece-wise constant time effect will be considered to accommodate possible non-linear time trends. This model will accommodate repeated patient visits and correlations within practices. All analyses will be carried out based on the intention to treat principle, which means that the treatment mode variable ($X_{ijt})$ is determined by the scheduled assignment of intervention implementation based on randomization, but not by the actual timing of implementation.

Given the probability of the treatment effect varying over time, we will consider the treatment effect, θ, as a function of post-intervention time, τ, such that $\theta=\theta(\tau)$. This is subsequently formulated as a random effect, with $\theta=\theta\left( \right)= \theta_{0}+\theta_{\tau}, \theta_{\tau}\sim N\left( 0, \sigma_{\theta}^{2} \right)$;[^163^](#_ENREF_163) in this case, $\theta_{\tau}$follows a normal distribution with mean zero and variance $\sigma_{\theta}^{2}$. We will apply Empirical Bayes estimates to assess the fluctuating treatment effect at various post-intervention times. We will estimate the probability of AWV completion between baseline and 12-months post-implementation, as well as between baseline and maintenance periods (18- and 24-months post-implementation, respectively).

Practices will have a heterogeneous composition of patient characteristics that have been associated with AWV and preventive services use. We will include these factors as covariates when comparing the change in AWV and preventive services use between pre- and post-intervention groups: age, gender, race (Asian, Black/African-American, White, Other), ethnicity (Hispanic, non-Hispanic), insurance coverage (fee-for-service Medicare, Medicare Advantage), and # of office visits per year. We will also include practice setting (community-based, academic, safety net), practice size, and percentage of racial/ethnic minority patients. We will also conduct subgroup analysis to examine heterogeneity of treatment effects by patient and practice characteristics, such as patient race/ethnicity, number of chronic conditions, and practice type (community-based, academic, safety net) on the effect of the intervention.

***Sensitivity analysis.***

Our main analysis will be a mixed effects logistic regression model with a linear secular time trend. As a sensitivity analysis, we will also explore a piece-wise constant time trend to accommodate a potential non-linear time trend. In addition, we will examine possible time-treatment interaction, to see if the treatment effect increases or decreases over time.

***Secondary objective (examine effect of PT-AWV intervention on reducing racial/ethnic disparities in AWV utilization)***: For each practice and organization, we will summarize use of AWVs by patient race/ethnicity at baseline and every 6 months (up until month 48 of the study) using descriptive statistics. We will also use run charts to illustrate and compare monthly AWV utilization by patient race/ethnicity. **Linear mixed effects models** will be used to directly estimate the percentage change in AWVs and in disparity reduction. The primary covariates in these models are race (Asian, Black/African-American, White, Other) and ethnicity. These models will include fixed effects of treatment, time, treatment-race/ethnicity interaction and patient and practice-level covariates. Other covariates will include: age; gender; insurance coverage (fee-for-service Medicare, Medicare Advantage); # of office visits per year; and practice setting, size, and % of racial/ethnic minority patients. Patients and practices are considered as random effects and piece-wise constant time effect will be constructed to compare the racial/ethnicity disparities in AWV use before and after the intervention, based on the estimates of fixed effects.

***Missing data.*** Mixed effects models accommodate the imbalance of patient data, assuming that they are missing at random. Based on our preliminary data, we expect few missing data for patient age, gender, insurance and number of office visits per year. For missing data on race, we will perform analyses with missing race as a variable and also will impute the race/ethnicity variable.

#### 10.4.3 Analysis of the Secondary Endpoints

The secondary endpoints are utilization of the individual preventive health services (listed in [**Section 4** **Table 2**](#_4_OBJECTIVES_AND)). For each practice and organization, we will summarize use of individual preventive health services at baseline and every 6 months until the end of the study using descriptive statistics. We will also use run charts to illustrate the monthly percentage of eligible patients who utilized each preventive health service assessed.

**Linear mixed effects models** will be used to assess the effect of the intervention on these secondary endpoints (**Table 7**). We will perform similar analyses to those described in [**section 10.4.2**](#_10.4.2_Analysis_of).

| **Table 7. Patient cohorts, secondary endpoints, and timing of measurement** | | |
| --- | --- | --- |
| **Cohort** | **Endpoints** | **Time Frame** |
| Of Medicare patients aged 50+, with 1 or more visits in the past 12 months, not deceased: those meeting additional eligibility requirements for each preventive service listed in **Table 4** (e.g., age, gender). * | Utilization of each individual preventive health service listed in **Table 4** (% of patients up to date for each preventive health service) | Monthly |
| ***** HEDIS / CMS inclusion and exclusion criteria will be utilized, as applicable | | |

#### 10.4.4 Analysis of the Tertiary Endpoints

Tertiary endpoints relate to evaluating intervention adoption and implementation by describing facilitators and barriers to adoption through analysis of semi-structured interviews and clinician surveys.

Dr. Tarn will guide analysis of semi-structured interviews, with the support of a graduate-level research team member with qualitative research method expertise. Two members of the research team will review transcripts, both independently and together to inductively generate codes relevant to pre-defined measures guided by the RE-AIM framework.[^164-167^](#_ENREF_164) Co-investigators and patient partners will participate in selected analyses to ensure the trustworthiness and robustness of the findings. We will use a template coding approach[^168^](#_ENREF_168) to efficiently identify and segment data relevant to pre-defined measures guided by the RE-AIM framework. Case-based matrices[^169^](#_ENREF_169) will help us to refine and synthesize our understanding of modifiable reasons that emerge as obstacles for implementing and sustaining the PT-AWV intervention, strategies for mitigating obstacles, and practice strengths that facilitate implementation and sustainability. Analyses will compare and contrast themes raised in different practice settings. Analyses will be done in ATLAS.ti, a software program for analyzing qualitative data. Clinician survey responses will be tabulated to determine the intervention components being used. The investigate team will use content analysis to analyze free-text responses to survey questions on barriers and facilitators of AWV completion.

#### 10.4.5 Safety Analyses

Not applicable

#### 10.4.6 Baseline Descriptive Statistics

Basic descriptive statistics will be generated to describe including number and type of clinicians in participating practices, and to describe demographics of patients eligible for AWVs: age (means with standard deviations; range), gender (percentages), race/ethnicity (percentages). For each practice and organization, we will summarize use of AWVs, the composite preventive health services score, and individual preventive health services at baseline (Month 0) and every 6 months until the end of the study.

#### 10.4.7 Planned Interim Analyses

Not applicable

#### 10.4.8 Sub-Group Analyses

Subgroup analyses will compare patients with fee-for-service versus Medicare Advantage insurance.

#### 10.4.9 Tabulation of individual Participant Data

Individual participant data will not be listed by measure and time point.

#### 10.4.10 Exploratory Analyses

Exploratory analyses will use ***propensity score analyses*** to examine the effect of AWV use on each of the outcomes. By estimating the probability of receiving an AWV in the post-intervention period, these analyses can account for potential differences among patients who do and do not receive AWVs. Those utilizing AWVs may differ from other patients in substantial ways that might affect the outcomes measured.

## 11 REGULATORY, ETHICAL, AND STUDY OVERSIGHT CONSIDERATIONS

### 11.1 Informed Consent Process

#### 11.1.1 Consent/Assent Procedures and Documentation

***Population cohort***. A waiver of informed consent was obtained for data extractions to assess outcome measures for the population cohort, as it is not feasible to obtain informed consent for all patients in this cohort. Patients will have no direct contact with the study investigators, and the investigators will receive only limited datasets for analyses.

***Semi-structured interview cohort***. A waiver of signed informed consent was obtained for semi-structured interviews with patients, clinicians, and staff. These interviews are low risk and written informed consent would be difficult to obtain as the interviews will not occur in-person.

Information sheets and oral consent scripts have been approved by the UCLA Institutional Review Board (IRB). Participants will be emailed the information sheet for review prior to the interview. For those who are unable to receive the information sheet by email or who did not receive it, a team member will read the oral consent script to participants. This will be done either when the interview is scheduled or immediately before the telephone interview begins.

Both the information sheet and oral consent script contain explanations of the purpose, procedures, and potential risks of the study and of their rights as research participants. Participants will be informed that participation is voluntary and that they may withdraw from the study at any time, without prejudice, and that the quality of their medical care will not be adversely affected if they decline to participate in this study.

Before the interview begins, the interviewer will ask participants if they have questions about the information sheet / oral consent script, and will answer any questions that may arise.

The research is no greater than minimal risk and only involves procedures for which written consent is normally not required outside the research context.

***Clinician survey cohort.*** A waiver of signed informed consent was obtained for clinician surveys. Information regarding the survey will be presented to clinicians immediately before they begin taking the survey (as the first page of the electronic survey). This information will contain explanations of the purpose, procedures, and potential risks of the study and of their rights as research participants. Clinicians will be informed that participation is voluntary and that they may withdraw from the study at any time.

#### 11.1.2 Consent for minors when they reach the age of majority

Not applicable

#### 11.1.3 Considerations for Consent of NIH staff, or family members of study team members

Not applicable

#### 11.1.4 Consent of Subjects who are, or become, decisionally impaired

Adults unable to provide consent are excluded from enrolling in semi-structured interviews.

### 11.2 Study Discontinuation and Closure

This study may be temporarily suspended or prematurely terminated if there is sufficient reasonable cause. Written notification, documenting the reason for study suspension or termination, will be provided by the suspending or terminating party to the investigator, funding agency, and regulatory authorities. If the study is prematurely terminated or suspended, the Principal Investigator (PI) will promptly inform study practices, the Institutional Review Board (IRB), and sponsor/funding agency and will provide the reason(s) for the termination or suspension. Study participants will be contacted, as applicable, and be informed of changes to the study visit schedule.

Circumstances that may warrant termination or suspension include, but are not limited to:

- Determination of unexpected, significant, or unacceptable risk to participants
- Demonstration of efficacy that would warrant stopping
- Insufficient compliance of study staff to the protocol requirements
- Data that are not sufficiently complete and/or evaluable
- Determination that the primary endpoint has been met
- Determination of futility

The study may resume once concerns about safety, protocol compliance, and data quality are addressed, and satisfy the NIA or other relevant regulatory or oversight bodies (OHRP, DSMB).

### 11.3 Confidentiality and Privacy

Participant confidentiality and privacy is strictly held in trust by the participating investigators, their staff, the safety and oversight monitor(s), and the sponsor(s) and funding agency. This confidentiality is extended to the data being collected as part of this study. Data that could be used to identify a specific study participant will be held in strict confidence within the research team. No personally identifiable information from the study will be released to any unauthorized third party without prior written approval of the sponsor/funding agency.

All research activities will be conducted in as private a setting as possible.

***Population cohort data.*** Study participant research data for the population cohort, which is for purposes of statistical analysis and scientific reporting, will be transmitted as limited datasets to UCLA, and will be stored at UCLA. The study data management systems used by the DARTNet Institute, AAFP NRN, and UCLA research staff will be secured and password protected.

***Semi-structured interview data.*** For semi-structured interview participants, contact information will be securely stored on a secure network server at UCLA for internal use during the study or on a UCLA-approved HIPAA-compliant cloud storage solution such as BOX. At the end of the study, all records will continue to be kept in a secure location for as long a period as dictated by the reviewing IRB, Institutional policies, or sponsor/funding agency requirements, after which patient contact information will be deleted. Audio recordings and de-identified transcripts of audio recordings will be maintained indefinitely.

***Survey data.*** Survey data will be securely stored on UCLA Health Qualtrics or REDCap, both of which are HIPAA-compliant survey platforms.

#### 11.3.1 Measures Taken to Ensure Confidentiality of Data Shared per the NIH Data Sharing Policies

It is NIH policy that the results and accomplishments of the activities that it funds should be made available to the public (see <https://grants.nih.gov/policy/sharing.htm>). The PI will ensure all mechanisms used to share data will include proper plans and safeguards for the protection of privacy, confidentiality, and security for data dissemination and reuse (e.g., all data will be thoroughly de-identified and will not be traceable to a specific study participant). Plans for archiving and long-term preservation of the data will be implemented, as appropriate.

#### 11.3.2 Certificate of Confidentiality

To further protect the privacy of study participants, the Secretary, Health and Human Services (HHS), has issued a Certificate of Confidentiality (CoC) to all researchers engaged in biomedical, behavioral, clinical or other human subjects research funded wholly or in part by the federal government.  Recipients of NIH funding for human subjects research are required to protect identifiable research information from forced disclosure per the terms of the NIH Policy (see <https://humansubjects.nih.gov/coc/index>). As set forth in [45 CFR Part 75.303(a)](https://www.ecfr.gov/cgi-bin/text-idx?SID=f3e9328bbbd5aabe8e639ca48dcbcc7f&mc=true&node=se45.1.75_1303&rgn=div8) and [NIHGPS Chapter 8.3](https://grants.nih.gov/grants/policy/nihgps/HTML5/section_8/8.3_management_systems_and_procedures.htm), recipients conducting NIH-supported research covered by this Policy are required to establish and maintain effective internal controls (e.g., policies and procedures) that provide reasonable assurance that the protocol is managed in compliance with Federal statutes, and regulations. It is the NIH policy that investigators and others who have access to research records will not disclose identifying information except when the participant consents or in certain instances when federal, state, or local law or regulation requires disclosure. NIH expects investigators to inform research participants of the protections and the limits to protections provided by a Certificate issued by this Policy.

### 11.4 Future use of Stored Specimens and Data

Data collected for this study will be analyzed and stored at the University of California, Los Angeles. After the study is completed, de-identified, archived data will be transmitted to and stored at Dryad (through the University of California), for use by other researchers including those outside of the study. Permission to transmit data to Dryad will be included in data use agreements with participating practices.

### 11.5 Safety Oversight

Safety oversight will be under the direction of a Data and Safety Monitoring Board (DSMB) composed of individuals with appropriate expertise, including a geriatrician, a health services researcher with informatics expertise, and a statistician. Members of the DSMB will be independent from the study conduct and free of conflict of interest. The DSMB will meet at least annually to assess study progress, conduct, and safety data. At this time, each data element that the DSMB needs to assess will be clearly defined. The DSMB will provide its input to the National Institute on Aging / National Institutes of Health staff.

### 11.6 Clinical Monitoring

Not applicable

### 11.7 Quality Assurance and Quality Control

All sites will follow a common quality management plan. Quality control (QC) procedures will be implemented as follows:

**Intervention Fidelity** —Procedures for ensuring fidelity of intervention delivery and collection of semi-structured interview and survey data are described in the **Manual of Procedures (MOP)** and in **Section 7.2.1, Interventionist Training and Tracking**.

**Metrics.** The DARTNet Institute will perform quality checks and data cleaning using proprietary software and systems, and will format data into OMOP v6 data models with SNOMED and RxNorm codes. Data quality will be enhanced through a series of programmed data quality checks that automatically detect out-of-range or anomalous data.

**Protocol Deviations** – The study team will review protocol deviations on an ongoing basis and will implement corrective actions when the quantity or nature of deviations are deemed to be at a level of concern.

### 11.8 Data Handling and Record Keeping

#### 11.8.1 Data Collection and Management Responsibilities

***Semi-structured interviews***

Data collection activities include collecting qualitative audio-recordings of semi-structured interviews. These interviews will be performed by the Principal Investigator and/or a research associate or other team member trained in qualitative research methods. The interviewer(s) will save digital audio recording files on a secure network server or on UCLA Health BOX on the day of collection.

The **MOP** describes procedures for collecting interview audio recordings. There are no case report forms for this study.

***Surveys***

Surveys will be sent to clinicians using personalized links through UCLA Health Qualtrics or REDCap. Downloaded data will be saved on a secure network server or on UCLA Health BOX and will be de-identified. The **MOP** describes procedures for survey distribution.

***EHR data extractions***

Data collection and sharing of data extractions will be carried out in accordance with HIPAA regulations and will follow the execution of Data Use Agreements between UCLA and each of the participating practices, as well as approvals from the UCLA Institutional Review Board. UCLA will also establish a Data Use Agreement with DARTNet to allow for transfer of limited data from UCLA to DARTNet. The DARTNet institute will establish Business Associate Agreements with sites from which they will perform data extractions. Only data relevant to this study will be extracted and transferred. Adequate provisions will be established to ensure the security of the data and to maintain its confidentiality. All data will be protected by HIPAA-compliant IT systems and will be uploaded on HIPAA-compliant cloud storage systems.

Data coordinating center. The DARTNet Institute will serve as the data coordinating center. DARTNet will receive limited data from the participating organizations or will establish an agreement to extract data from the organization’s EHR system. A list of data elements to be extracted can be found in [Section 13 Appendix](#_13_APPENDIX).

DARTNet Data Environments**.** DARTNet maintains HIPAA compliant data in two different environments. Physical machines are housed in a HIPAA-compliant server room maintained by the central IT department of the University of Colorado, Denver. Access is monitored 24 hours a day, 7 days a week with key card monitoring and video recording. All DARTNet servers are on their own sub-network behind a second firewall maintained by DARTNet, along with a second intrusion detection system also maintained by DARTNet. Electronic access is through the University of Colorado, Denver VPN and access to all servers requires duo-factor authentication. All servers are scanned at least monthly for vulnerabilities. Third party risk assessments and intrusion tests are conducted annually. All servers are regularly patched. They are also encrypted at rest and selected systems overlay database level encryption on the disk level encryption system.

DARTNet also maintains a HIPAA-compliant cloud-based computing center. This center is physically hosted by Amazon Web Server in the Portland, OR area. All systems are encrypted and all access is through individual user accounts using secure portals.

AAFP NRN Data Environments. The American Academy of Family Physicians National Research Network (AAFP NRN) has a secured, HIPAA compliant server on the University of Colorado – Denver system to conduct data management and analyses on data that requires HIPAA compliance. This network is the same network that houses DARTNet Institute’s environment, though on different servers. On this server, a SAS license is provided for data management and analysis. The data used in this project will be stored on this server and within DARTNet Institute’s Egnyte system. Egnyte is also a HIPAA compliant storage system and file transfer system.

The DARTNet Institute will extract or receive data from practices and process it through their Egnyte system. The AAFP NRN will access data from the secured HIPAA compliant server after initial processing by DARTNet. After downloading, the AAFP NRN will clean the data and create limited analytic datasets that will be transferred to UCLA for analysis. Technical safeguards will be in place to protect storage and transmission, including end-to-end encryption and firewall protection.

UCLA Health Data Environments. UCLA will receive and maintain limited datasets from the AAFP NRN. The AAFP NRN will save these files directly into a HIPAA-compliant cloud-based computing system such as BOX storage, where the data will be maintained. Technical safeguards will be in place to protect storage and transmission, including end-to-end encryption and firewall protection. Electronic access will be via a unique user identification and password.

#### 11.8.2 Study Records Retention

De-identified quantitative data on the population cohort of patients will be placed in a data repository and maintained indefinitely.

Audio recordings of semi-structured interviews, de-identified transcripts, and survey data will be maintained indefinitely. Patient contact information will be deleted at the end of the study. Permission is not required prior to the destruction of this information.

### 11.9 Protocol Deviations and Non-Compliance

It is the responsibility of the investigator to use continuous vigilance to identify and report deviations and/or non-compliance to the UCLA Institutional Review Board. All deviations must be addressed in study documents, reported to the National Institute on Aging Program Official and to the UCLA Institutional Review Board. The investigator is responsible for knowing and adhering to the reviewing IRB requirements.

#### 11.9.1 NIH Definition of Protocol Deviation

A protocol deviation is any changed, divergence, or departure from the IRB-approved research protocol.

- Major deviations: Deviations from the IRB approved protocol that have, or may have the potential to, negatively impact the rights, welfare or safety of the subject, or to substantially negatively impact the scientific integrity or validity of the study.
- Minor deviations: Deviations that do not have the potential to negatively impact the rights, safety or welfare of subjects or others, or the scientific integrity or validity of the study.

### 11.10 Publication and Data Sharing Policy

#### 11.10.1 Human Data Sharing Plan

This study will be conducted in accordance with the following publication and data sharing policies and regulations:

National Institutes of Health (NIH) Public Access Policy, which ensures that the public has access to the published results of NIH funded research. It requires scientists to submit final peer-reviewed journal manuscripts that arise from NIH funds to the digital archive PubMed Central upon acceptance for publication.

This study will comply with the NIH Data Sharing Policy and Policy on the Dissemination of NIH-Funded Clinical Trial Information and the Clinical Trials Registration and Results Information Submission rule. As such, this trial will be registered at ClinicalTrials.gov, and results information from this trial will be submitted to ClinicalTrials.gov. In addition, every attempt will be made to publish results in peer-reviewed journals. Data from this study may be requested by other researchers after the completion of the primary endpoint by making a request through the University of California Curation Center (UC3), which maintains the Dryad publishing platform.

#### 11.10.2 Genomic Data Sharing Compliance

Not applicable

### 11.11 Collaborative Agreements

#### 11.11.1 Agreement Type

Not applicable

### 11.12 Conflict of Interest Policy

The independence of this study from any actual or perceived influence is critical. Therefore, any actual conflict of interest of persons who have a role in the design, conduct, analysis, publication, or any aspect of this trial will be disclosed and managed. Furthermore, persons who have a perceived conflict of interest will be required to have such conflicts managed in a way that is appropriate to their participation in the design and conduct of this trial. The study leadership in conjunction with the National Institute on Aging has established policies and procedures for all study group members to disclose all conflicts of interest and will establish a mechanism for the management of all reported dualities of interest.

## 12 ABBREVIATIONS

| AAFP NRN | American Academy of Family Physicians National Research Network |
| --- | --- |
| ACIP | Advisory Committee on Immunization Practices |
| AE | Adverse Event |
| AWV | Annual Wellness Visit |
| CDC | Centers for Disease Control and Prevention |
| CFR | Code of Federal Regulations |
| DSMB | Data Safety Monitoring Board |
| EHR | Electronic health record |
| FDA | Food and Drug Administration |
| GCP | Good Clinical Practice |
| HIPAA | Health Insurance Portability and Accountability Act |
| HHS | Health and Human Services |
| ICC | Intra-class correlation coefficient |
| ICH | International Conference on Harmonisation |
| IPPE | Initial Preventive Physical Examination |
| IRB | Institutional Review Board |
| MOP | Manual of Procedures |
| NCT | National Clinical Trial |
| NIH | National Institutes of Health |
| OHRP | Office for Human Research Protections |
| PI | Principal Investigator |
| PT-AWV | Practice-Tailored Annual Wellness Visit |
| QC | Quality Control |
| SAE | Serious Adverse Event |
| SOA | Schedule of Activities |
| UCLA | University of California, Los Angeles |
| UP | Unanticipated Problem |
| US | United States |
| USPSTF | United States Preventive Services Task Force |

##

## 13 APPENDIX

**Data Extraction Elements**

| Metric name | Cohort | Metric Description | Value set (Inclusion / Numerator) | Value set (Exclusion) |
| --- | --- | --- | --- | --- |
| Patient Demographics | Entire cohort | N/A | Month and Year of birth  Gender  Race  Ethnicity | None |
| Annual Wellness Visit | Entire cohort | Had IPPE or AWV (initial or subsequent) within past 12 months | G0422  G0438  G0439 | None |
| Influenza | Entire cohort | % of patients up to date on influenza vaccine  (received on or between July 1 of year prior to measurement period to June 30 of measurement period OR prior influenza adverse reaction) | imm_flu  alg_imm_flu | None |
| Tetanus | Entire cohort | % of patients up to date on tetanus vaccine  (received at least one Td or Tdap vaccine between 9 years prior to start of measurement period and end of measurement period or prior adverse reaction) | imm_td  imm_tdap  imm_tetanus  alg_imm_dtap  alg_imm_tdap  alg_imm_tetanus | None |
| Herpes Zoster | 50 years of age and older | % of patients up to date on zoster vaccine  (received at least one dose of zostavax or 2 doses of shingrix at least 28 days apart or prior adverse reaction) | imm_zoster_shingrix2  imm_zoster_zostavax1  alg_imm_zoster | dx_imm_zoster_contraindications |
| Pneumococcal | 66 years of age and older | % of patients up to date on pneumococcal vaccine (Received at least one dose of pcv-20 or pcv-23 on or after 60th birthday before or during measurement period or prior adverse reaction) | imm_pcv  imm_ppsv23  imm pcv20  alg_imm_pcv  alg_imm_pcv23  alg immpcv20 | None |
| Cervical Cancer | Women aged 24-64 years | % of patients completing cervical cancer screening  Women 24-64 years of age who had cervical cytology during the measurement year or the two years prior to the measurement year OR  Women 30-64 years of age who had cervical HPV testing during the measurement year or the 4 years prior to the measurement year AND who were 30 years or older on the date of the test. | gender_female  lab_pap  lab_hpv_screen  lab_pap_abnormal | doc_hospice  dx_palliative  pmh_hysterectomy_vaginal |
| Mammogram | Women aged 52-74 years | % women 50-74 who had a mammogram | gender_female  proc_mammogram  dx_breast_ca_screen | pmh_mastectomy_bilateral  pmh_mammogram_unilateral  doc_hospice  dx_palliative  dx_frailty  dx_advanced_illness |
| Colon cancer | 51-75 years of age | % of patients 50-75 with appropriate colon cancer screening  1) Fecal occult blood test within past 12 months  2) Flex sig within the past 5 years  3) Colonoscopy within the past 10 years  4) CT colonography within the past 5 years  5) FIT-DNA test within the past 3 years | lab_fobt  lab_ifobt_fit  proc_flexible_sigmoidoscopy  proc_colonoscopy  proc_colon_ca_scrn_adenoma  proc_ct_colonography  lab_stool_dna_cologuard | pmh_colorectal_cancer  pmh_total_colectomy  doc_hospice  dx_palliative  dx_frailty  dx_advanced_illness |
| Prostate specific antigen | Men aged 70 years and older | % men 70 and over who were screened for prostate cancer  PSA based screening test performed during the measurement year | gender_male  lab_psa  lab_psa_free | pmh_cancer_prostate  *PSA test in the year prior to the measurement year with result > 4.0 ng/mL (or indication of abnormal test)*  lab_psa  Lab_elevated_psa  *Prescription for 5-alpha reductase inhibitor*  med_a_reduc_inhibit |
| Ultrasound for abdominal aortic aneurysm | Men aged 66-75 years with history of tobacco use  current or past smoker:  dx_tobacco_use  med_tobacco_cessation  ref_smoking_cessation_clinic  soc_past_smoker  soc_smoker  soc_smoker_heavy  soc_smoker_heavy_former  soc_smoker_light  soc_smoker_light_former  soc_smoker_moderate  soc_smoker_moderate_former  soc_smoker_pack_years soc_smoker_years_total  soc_tobacco_cessation  soc_tobacco_current soc_tobacco_past | % men 65-75 who have ever smoked who received 1-time screening for AAA  Abdominal US or other abdominal imaging performed on or after 65th birthday or during measurement period  OR presence of at least 1 AAA in past | gender_male  proc_aaa_screen  proc_abdominal_ct  proc_abdominal_mri  proc_abdominal_us  dx_aaa | soc_non_smoker  soc_tobacco_non_never  doc_hospice  dx_palliative  *within past 6 months*  proc_chemotherapy  *within past 3 months*  doc_nursing_facility |
| Osteoporosis | Women aged 66-75 years of age | % of women 65-75 years of age who received osteoporosis screening  One or more osteoporosis screening tests on or between the patient's 65th birthday and measurement period | gender_female  proc_bmd | dx_osteoporosis  pmh_osteoporosis  med_osteo_prolia  med_osteo_reclast  med_bisphosphonate  med_osteo_tymlos  doc_hospice  dx_palliative  *Exclude if aged 66 and older with BOTH frailty and advanced illness during measurement period*  dx_frailty  dx_advanced_illness |
| Advance Care Planning | Aged 66 and older | % of patients aged 66 and older with documentation of advance care planning | soc_adv_directive | N/A |
| Hepatitis C | Aged 18-79 years of age | % of patients who received one-time hepatitis C screening | lab_hepatitis_c_ab  lab_hepatitis_c_rna  lab_hepatitis_c_genotype  lab_hepatitis_c_screen | dx_hepatitis_c  dx_hepatitis_c_acute  dx_cirrhosis  dx_hepat_carcinoma  dx_decomp_cirrhosis  dx_esophageal_var  dx_hepatic_encep  dx_ascites  doc_hospice  dx_palliative |
| Depression | 1) Entire cohort    2) Those in cohort 1 with positive finding for depression in the past 12 months  dx_depression  dx_depression_major  doc_phq9_gt9 | 1) % of patients who were screened for depression in the past 12 months  2) For those screening positive for depression, % that received follow-up care within 30 days of a positive screen | doc_depression_score  doc_depression_screen  doc_depression_screen_maternal  doc_phq2_score  doc_phq2_screen  doc_phq9_score  doc_phq9_screen  doc_depression_plan  med_antidepressant  med_antidepressant_monotherapy  ref_mental_hlth_psych  ref_behavioral_health_clinic  visit_behavioral_health  ibh_med_antidepressant | dx_bipolar_disorder  *Depression that starts during past 12 months*  dx_depression  dx_depression_major  dx_mental_beh_dis  doc_hospice  dx_palliative |
| Alcohol use | 1) Entire cohort  2) Those in cohort 1 with positive finding for unhealthy alcohol use  soc_alcohol_use_disorder  dx_alcohol_related_disorders  doc_audit_score | 1) % of patients who were screened for alcohol use one or more times within the past 24 months  2) % of patients in Cohort 1 who  screened positive and received an intervention on or up to 60 days after the date of the first positive screen (61 days total) | doc_audit_screen  doc_audit_score  soc_alcohol_screen  soc_alcohol_use_current  soc_alcohol_use_former  soc_alcohol_use_disorder  soc_alcohol_use_none  med_antialcohol  soc_alcohol_counsel_plan  soc_substance_abuse_counsel  ref_alcohol_rehab  ref_drug_addiction_mgmt  ibh_med_antialcohol | soc_alcohol_use_disorder  dx_alcohol_related_disorders  dx_dementia  doc_hospice  dx_palliative  *Exclusion only for cohort 2*  soc_alcohol_use_none |
| Tobacco use | 1) Entire cohort  2) All patients in cohort 1 identified as tobacco users  3) Entire cohort | 1) Screened for tobacco use at least once within 24 months  2) % of patients in Cohort 1 who were identified as tobacco users who received tobacco cessation counseling  3) % of patients who were screened for tobacco use within 24 months AND if screened positive, received intervention on or up to 60 days after the date of the first positive screen (61 days total) | dx_tobacco_use  soc_non_smoker  soc_past_smoker  soc_smoker  soc_smoker_ecigarette  soc_smoker_heavy  soc_smoker_heavy_former  soc_smoker_light  soc_smoker_light_former soc_smoker_moderate soc_smoker_moderate_former soc_smoker_pack_years  soc_smoker_years_total  soc_tobacco_cessation  soc_tobacco_current  soc_tobacco_non_never  soc_tobacco_past  soc_tobacco_screen  soc_tobacco_unspecified  med_tobacco_cessation  ref_smoking_cessation_clinic  soc_tobacco_cessation | doc_hospice  dx_palliative |

## REFERENCES

1. Zeiger TM, Thatcher EJ, Kirpekar S, Coran JJ, Topalsky G, Zarach MJD, Cox DA, Schario ME, Fuller KA, Upton PM, et al. Achieving Large-Scale Quality Improvement in Primary Care Annual Wellness Visits and Hierarchical Condition Coding. *Journal of General Internal Medicine*. 2022. doi: 10.1007/s11606-021-07323-1

2. AARP Public Policy Institute. Racial and ethnic disparities in influenza and pneumococcal immunization rates among Medicare beneficiaries. AARP Insight on the Issues, October 2008. <http://assets.aarp.org/rgcenter/health/i12_flu.pdf>. 2008. Accessed July 16.

3. Wee CC, McCarthy EP, Phillips RS. Factors associated with colon cancer screening: the role of patient factors and physician counseling. *Prev Med*. 2005;41:23-29. doi: 10.1016/j.ypmed.2004.11.004

4. Seeff LC, Nadel MR, Klabunde CN, Thompson T, Shapiro JA, Vernon SW, Coates RJ. Patterns and predictors of colorectal cancer test use in the adult U.S. population. *Cancer*. 2004;100:2093-2103. doi: 10.1002/cncr.20276

5. Bluestein D, Diduk-Smith R, Jordan L, Persaud K, Hughes T. Medicare Annual Wellness Visits: How to Get Patients and Physicians on Board. *Fam Pract Manag*. 2017;24:12-16.

6. Beran MS, Craft C. Medicare annual wellness visits. Understanding the patient and physician perspective. *Minn Med*. 2015;98:38-41.

7. Shokar NK, Carlson CA, Weller SC. Factors associated with racial/ethnic differences in colorectal cancer screening. *J Am Board Fam Med*. 2008;21:414-426. doi: 10.3122/jabfm.2008.05.070266

8. Schwartz JS, Lewis CE, Clancy C, Kinosian MS, Radany MH, Koplan JP. Internists' practices in health promotion and disease prevention. A survey. *Ann Intern Med*. 1991;114:46-53. doi: 10.7326/0003-4819-114-1-46

9. Winston CA, Wortley PM, Lees KA. Factors associated with vaccination of medicare beneficiaries in five U.S. communities: Results from the racial and ethnic adult disparities in immunization initiative survey, 2003. *J Am Geriatr Soc*. 2006;54:303-310. doi: 10.1111/j.1532-5415.2005.00585.x

10. Rim SH, Zittleman L, Westfall JM, Overholser L, Froshaug D, Coughlin SS. Knowledge, attitudes, beliefs, and personal practices regarding colorectal cancer screening among health care professionals in rural Colorado: a pilot survey. *J Rural Health*. 2009;25:303-308. doi: 10.1111/j.1748-0361.2009.00234.x

11. Guerra CE, Schwartz JS, Armstrong K, Brown JS, Halbert CH, Shea JA. Barriers of and facilitators to physician recommendation of colorectal cancer screening. *J Gen Intern Med*. 2007;22:1681-1688. doi: 10.1007/s11606-007-0396-9

12. Lind KE, Hildreth KL, Perraillon MC. Persistent Disparities in Medicare's Annual Wellness Visit Utilization. *Med Care*. 2019;57:984-989. doi: 10.1097/mlr.0000000000001229

13. Death Rates by Marital Status for Leading Causes of Death: United States, 2010–2019. In: National Vital Statistics Reports; 2022.

14. Heron M. Deaths: Leading causes for 2017. In: *National Vital Statistics Reports*. Hyattsville, MD: National Center for HealthStatistics; 2019.

15. Jodal HC, Helsingen LM, Anderson JC, Lytvyn L, Vandvik PO, Emilsson L. Colorectal cancer screening with faecal testing, sigmoidoscopy or colonoscopy: a systematic review and network meta-analysis. *BMJ open*. 2019;9:e032773-e032773. doi: 10.1136/bmjopen-2019-032773

16. Cardoso R, Guo F, Heisser T, Hackl M, Ihle P, De Schutter H, Van Damme N, Valerianova Z, Atanasov T, Májek O, et al. Colorectal cancer incidence, mortality, and stage distribution in European countries in the colorectal cancer screening era: an international population-based study. *Lancet Oncol*. 2021;22:1002-1013. doi: 10.1016/s1470-2045(21)00199-6

17. Gini A, Jansen EEL, Zielonke N, Meester RGS, Senore C, Anttila A, Segnan N, Mlakar DN, de Koning HJ, Lansdorp-Vogelaar I. Impact of colorectal cancer screening on cancer-specific mortality in Europe: A systematic review. *Eur J Cancer*. 2020;127:224-235. doi: 10.1016/j.ejca.2019.12.014

18. Zhang J, Cheng Z, Ma Y, He C, Lu Y, Zhao Y, Chang X, Zhang Y, Bai Y, Cheng N. Effectiveness of Screening Modalities in Colorectal Cancer: A Network Meta-Analysis. *Clin Colorectal Cancer*. 2017;16:252-263. doi: 10.1016/j.clcc.2017.03.018

19. Lin JS, Piper MA, Perdue LA, Rutter C, Webber EM, O'Connor E, Smith N, Whitlock EP. U.S. Preventive Services Task Force Evidence Syntheses, formerly Systematic Evidence Reviews. In: *Screening for Colorectal Cancer: A Systematic Review for the U.S. Preventive Services Task Force*. Rockville (MD): Agency for Healthcare Research and Quality (US); 2016.

20. Lin JS, Piper MA, Perdue LA, Rutter CM, Webber EM, O'Connor E, Smith N, Whitlock EP. Screening for Colorectal Cancer: Updated Evidence Report and Systematic Review for the US Preventive Services Task Force. *Jama*. 2016;315:2576-2594. doi: 10.1001/jama.2016.3332

21. Fitzpatrick-Lewis D, Ali MU, Warren R, Kenny M, Sherifali D, Raina P. Screening for Colorectal Cancer: A Systematic Review and Meta-Analysis. *Clin Colorectal Cancer*. 2016;15:298-313. doi: 10.1016/j.clcc.2016.03.003

22. Brenner H, Stock C, Hoffmeister M. Effect of screening sigmoidoscopy and screening colonoscopy on colorectal cancer incidence and mortality: systematic review and meta-analysis of randomised controlled trials and observational studies. *Bmj*. 2014;348:g2467. doi: 10.1136/bmj.g2467

23. Holme O, Bretthauer M, Fretheim A, Odgaard-Jensen J, Hoff G. Flexible sigmoidoscopy versus faecal occult blood testing for colorectal cancer screening in asymptomatic individuals. *Cochrane Database Syst Rev*. 2013:Cd009259. doi: 10.1002/14651858.CD009259.pub2

24. Elmunzer BJ, Hayward RA, Schoenfeld PS, Saini SD, Deshpande A, Waljee AK. Effect of flexible sigmoidoscopy-based screening on incidence and mortality of colorectal cancer: a systematic review and meta-analysis of randomized controlled trials. *PLoS Med*. 2012;9:e1001352. doi: 10.1371/journal.pmed.1001352

25. Whitlock EP, Lin J, Liles E, Beil T, Fu R, O'Connor E, Thompson RN, Cardenas T. U.S. Preventive Services Task Force Evidence Syntheses, formerly Systematic Evidence Reviews. In: *Screening for Colorectal Cancer: An Updated Systematic Review*. Rockville (MD): Agency for Healthcare Research and Quality (US); 2008.

26. Pignone M, Rich M, Teutsch SM, Berg AO, Lohr KN. Screening for colorectal cancer in adults at average risk: a summary of the evidence for the U.S. Preventive Services Task Force. *Ann Intern Med*. 2002;137:132-141. doi: 10.7326/0003-4819-137-2-200207160-00015

27. Pignone M, Saha S, Hoerger T, Mandelblatt J. Cost-effectiveness analyses of colorectal cancer screening: a systematic review for the U.S. Preventive Services Task Force. *Ann Intern Med*. 2002;137:96-104. doi: 10.7326/0003-4819-137-2-200207160-00007

28. Saquib N, Saquib J, Ioannidis JP. Does screening for disease save lives in asymptomatic adults? Systematic review of meta-analyses and randomized trials. *Int J Epidemiol*. 2015;44:264-277. doi: 10.1093/ije/dyu140

29. Zielonke N, Gini A, Jansen EEL, Anttila A, Segnan N, Ponti A, Veerus P, de Koning HJ, van Ravesteyn NT, Heijnsdijk EAM. Evidence for reducing cancer-specific mortality due to screening for breast cancer in Europe: A systematic review. *Eur J Cancer*. 2020;127:191-206. doi: 10.1016/j.ejca.2019.12.010

30. Nelson HD, Fu R, Cantor A, Pappas M, Daeges M, Humphrey L. Effectiveness of Breast Cancer Screening: Systematic Review and Meta-analysis to Update the 2009 U.S. Preventive Services Task Force Recommendation. *Ann Intern Med*. 2016;164:244-255. doi: 10.7326/m15-0969

31. Nelson HD, Cantor A, Humphrey L, Fu R, Pappas M, Daeges M, Griffin J. U.S. Preventive Services Task Force Evidence Syntheses, formerly Systematic Evidence Reviews. In: *Screening for Breast Cancer: A Systematic Review to Update the 2009 U.S. Preventive Services Task Force Recommendation*. Rockville (MD): Agency for Healthcare Research and Quality (US); 2016.

32. Pace LE, Keating NL. A systematic assessment of benefits and risks to guide breast cancer screening decisions. *Jama*. 2014;311:1327-1335. doi: 10.1001/jama.2014.1398

33. Nelson HD, Tyne K, Naik A, Bougatsos C, Chan B, Nygren P, Humphrey L. U.S. Preventive Services Task Force Evidence Syntheses, formerly Systematic Evidence Reviews. In: *Screening for Breast Cancer: Systematic Evidence Review Update for the US Preventive Services Task Force*. Rockville (MD): Agency for Healthcare Research and Quality (US); 2009.

34. Nelson HD, Tyne K, Naik A, Bougatsos C, Chan BK, Humphrey L. Screening for breast cancer: an update for the U.S. Preventive Services Task Force. *Ann Intern Med*. 2009;151:727-737, w237-742. doi: 10.7326/0003-4819-151-10-200911170-00009

35. Braithwaite D, Walter LC, Izano M, Kerlikowske K. Benefits and Harms of Screening Mammography by Comorbidity and Age: A Qualitative Synthesis of Observational Studies and Decision Analyses. *J Gen Intern Med*. 2016;31:561-572. doi: 10.1007/s11606-015-3580-3

36. Smith RA, Andrews KS, Brooks D, Fedewa SA, Manassaram-Baptiste D, Saslow D, Brawley OW, Wender RC. Cancer screening in the United States, 2018: A review of current American Cancer Society guidelines and current issues in cancer screening. *CA Cancer J Clin*. 2018;68:297-316. doi: 10.3322/caac.21446

37. Myers ER, Moorman P, Gierisch JM, Havrilesky LJ, Grimm LJ, Ghate S, Davidson B, Mongtomery RC, Crowley MJ, McCrory DC, et al. Benefits and Harms of Breast Cancer Screening: A Systematic Review. *Jama*. 2015;314:1615-1634. doi: 10.1001/jama.2015.13183

38. Oudkerk M, Devaraj A, Vliegenthart R, Henzler T, Prosch H, Heussel CP, Bastarrika G, Sverzellati N, Mascalchi M, Delorme S, et al. European position statement on lung cancer screening. *Lancet Oncol*. 2017;18:e754-e766. doi: 10.1016/s1470-2045(17)30861-6

39. Fabrikant MS, Wisnivesky JP, Marron T, Taioli E, Veluswamy RR. Benefits and Challenges of Lung Cancer Screening in Older Adults. *Clin Ther*. 2018;40:526-534. doi: 10.1016/j.clinthera.2018.03.003

40. Kotwal AA, Schonberg MA. Cancer Screening in the Elderly: A Review of Breast, Colorectal, Lung, and Prostate Cancer Screening. *Cancer J*. 2017;23:246-253. doi: 10.1097/ppo.0000000000000274

41. Usman Ali M, Miller J, Peirson L, Fitzpatrick-Lewis D, Kenny M, Sherifali D, Raina P. Screening for lung cancer: A systematic review and meta-analysis. *Prev Med*. 2016;89:301-314. doi: 10.1016/j.ypmed.2016.04.015

42. Hoffman RM, Atallah RP, Struble RD, Badgett RG. Lung Cancer Screening with Low-Dose CT: a Meta-Analysis. *J Gen Intern Med*. 2020;35:3015-3025. doi: 10.1007/s11606-020-05951-7

43. Hunger T, Wanka-Pail E, Brix G, Griebel J. Lung Cancer Screening with Low-Dose CT in Smokers: A Systematic Review and Meta-Analysis. *Diagnostics (Basel)*. 2021;11. doi: 10.3390/diagnostics11061040

44. Sadate A, Occean BV, Beregi JP, Hamard A, Addala T, de Forges H, Fabbro-Peray P, Frandon J. Systematic review and meta-analysis on the impact of lung cancer screening by low-dose computed tomography. *Eur J Cancer*. 2020;134:107-114. doi: 10.1016/j.ejca.2020.04.035

45. Guirguis-Blake JM, Beil TL, Senger CA, Whitlock EP. Ultrasonography screening for abdominal aortic aneurysms: a systematic evidence review for the U.S. Preventive Services Task Force. *Ann Intern Med*. 2014;160:321-329. doi: 10.7326/m13-1844

46. Ali MU, Fitzpatrick-Lewis D, Miller J, Warren R, Kenny M, Sherifali D, Raina P. Screening for abdominal aortic aneurysm in asymptomatic adults. *J Vasc Surg*. 2016;64:1855-1868. doi: 10.1016/j.jvs.2016.05.101

47. Guirguis-Blake JM, Beil TL, Senger CA, Coppola EL. Primary Care Screening for Abdominal Aortic Aneurysm: Updated Evidence Report and Systematic Review for the US Preventive Services Task Force. *JAMA*. 2019;322:2219-2238. doi: 10.1001/jama.2019.17021

48. Ying AJ, Affan ET. Abdominal Aortic Aneurysm Screening: A Systematic Review and Meta-analysis of Efficacy and Cost. *Ann Vasc Surg*. 2019;54:298-303.e293. doi: 10.1016/j.avsg.2018.05.044

49. Berical AC, Harris D, Dela Cruz CS, Possick JD. Pneumococcal Vaccination Strategies. An Update and Perspective. *Ann Am Thorac Soc*. 2016;13:933-944. doi: 10.1513/AnnalsATS.201511-778FR

50. Kraicer-Melamed H, O'Donnell S, Quach C. The effectiveness of pneumococcal polysaccharide vaccine 23 (PPV23) in the general population of 50 years of age and older: A systematic review and meta-analysis. *Vaccine*. 2016;34:1540-1550. doi: 10.1016/j.vaccine.2016.02.024

51. Cafiero-Fonseca ET, Stawasz A, Johnson ST, Sato R, Bloom DE. The full benefits of adult pneumococcal vaccination: A systematic review. *PLoS One*. 2017;12:e0186903. doi: 10.1371/journal.pone.0186903

52. Kim DK, Hunter P. Advisory Committee on Immunization Practices Recommended Immunization Schedule for Adults Aged 19 Years or Older - United States, 2019. *MMWR Morb Mortal Wkly Rep*. 2019;68:115-118. doi: 10.15585/mmwr.mm6805a5

53. Keating GM. Shingles (Herpes Zoster) Vaccine (Zostavax((R))): A Review in the Prevention of Herpes Zoster and Postherpetic Neuralgia. *BioDrugs*. 2016;30:243-254. doi: 10.1007/s40259-016-0180-7

54. Sanford M, Keating GM. Zoster vaccine (Zostavax): a review of its use in preventing herpes zoster and postherpetic neuralgia in older adults. *Drugs Aging*. 2010;27:159-176. doi: 10.2165/10489140-000000000-00000

55. Fiscella K, Dressler R, Meldrum S, Holt K. Impact of influenza vaccination disparities on elderly mortality in the United States. *Prev Med*. 2007;45:83-87. doi: 10.1016/j.ypmed.2007.03.007

56. Demicheli V, Jefferson T, Di Pietrantonj C, Ferroni E, Thorning S, Thomas RE, Rivetti A. Vaccines for preventing influenza in the elderly. *Cochrane Database Syst Rev*. 2018;2:Cd004876. doi: 10.1002/14651858.CD004876.pub4

57. Chan TC, Fan-Ngai Hung I, Ka-Hay Luk J, Chu LW, Hon-Wai Chan F. Effectiveness of influenza vaccination in institutionalized older adults: a systematic review. *J Am Med Dir Assoc*. 2014;15:226.e221-226.e226. doi: 10.1016/j.jamda.2013.10.008

58. Gross PA, Hermogenes AW, Sacks HS, Lau J, Levandowski RA. The efficacy of influenza vaccine in elderly persons. A meta-analysis and review of the literature. *Ann Intern Med*. 1995;123:518-527. doi: 10.7326/0003-4819-123-7-199510010-00008

59. Andrew MK, Bowles SK, Pawelec G, Haynes L, Kuchel GA, McNeil SA, McElhaney JE. Influenza Vaccination in Older Adults: Recent Innovations and Practical Applications. *Drugs Aging*. 2019;36:29-37. doi: 10.1007/s40266-018-0597-4

60. Tetanus surveillance --- United States, 2001-2008. *MMWR Morbidity and mortality weekly report*. 2011;60:365-369.

61. Adams DA, Thomas KR, Jajosky RA, Foster L, Sharp P, Onweh DH, Schley AW, Anderson WJ. Summary of Notifiable Infectious Diseases and Conditions - United States, 2014. *MMWR Morbidity and mortality weekly report*. 2016;63:1-152. doi: 10.15585/mmwr.mm6354a1

62. Kupronis BA, Richards CL, Whitney CG. Invasive pneumococcal disease in older adults residing in long-term care facilities and in the community. *J Am Geriatr Soc*. 2003;51:1520-1525. doi: 10.1046/j.1532-5415.2003.51501.x

63. Bricout H, Haugh M, Olatunde O, Prieto RG. Herpes zoster-associated mortality in Europe: a systematic review. *BMC Public Health*. 2015;15:466. doi: 10.1186/s12889-015-1753-y

64. Rimland D, Moanna A. Increasing incidence of herpes zoster among Veterans. *Clin Infect Dis*. 2010;50:1000-1005. doi: 10.1086/651078

65. Yawn BP, Saddier P, Wollan PC, St Sauver JL, Kurland MJ, Sy LS. A population-based study of the incidence and complication rates of herpes zoster before zoster vaccine introduction. *Mayo Clin Proc*. 2007;82:1341-1349. doi: 10.4065/82.11.1341

66. Sudore RL, Lum HD, You JJ, Hanson LC, Meier DE, Pantilat SZ, Matlock DD, Rietjens JAC, Korfage IJ, Ritchie CS, et al. Defining Advance Care Planning for Adults: A Consensus Definition From a Multidisciplinary Delphi Panel. *Journal of pain and symptom management*. 2017;53:821-832.e821. doi: 10.1016/j.jpainsymman.2016.12.331

67. Lum HD, Sudore RL, Bekelman DB. Advance care planning in the elderly. *Med Clin North Am*. 2015;99:391-403. doi: 10.1016/j.mcna.2014.11.010

68. Brinkman-Stoppelenburg A, Rietjens JA, van der Heide A. The effects of advance care planning on end-of-life care: a systematic review. *Palliative medicine*. 2014;28:1000-1025. doi: 10.1177/0269216314526272

69. Houben CHM, Spruit MA, Groenen MTJ, Wouters EFM, Janssen DJA. Efficacy of advance care planning: a systematic review and meta-analysis. *J Am Med Dir Assoc*. 2014;15:477-489. doi: 10.1016/j.jamda.2014.01.008

70. Centers for Disease Control and Prevention. The State of Aging and Health in America 2013. In: Atlanta, GA: Centers for Disease Control and Prevention, US Dept of Health and Human Services; 2013.

71. Rubin R. Filling the Gaps in Preventive Care Services for Older Adults. *JAMA*. 2015;313:1604-1606. doi: 10.1001/jama.2015.2369

72. Williams WW, Lu PJ, O'Halloran A, Kim DK, Grohskopf LA, Pilishvili T, Skoff TH, Nelson NP, Harpaz R, Markowitz LE, et al. Surveillance of Vaccination Coverage among Adult Populations - United States, 2015. *MMWR Surveill Summ*. 2017;66:1-28. doi: 10.15585/mmwr.ss6611a1

73. Lu PJ, Hung MC, Srivastav A, Grohskopf LA, Kobayashi M, Harris AM, Dooling KL, Markowitz LE, Rodriguez-Lainz A, Williams WW. Surveillance of Vaccination Coverage Among Adult Populations -United States, 2018. *MMWR Surveill Summ*. 2021;70:1-26. doi: 10.15585/mmwr.ss7003a1

74. Siegel RL, Miller KD, Fedewa SA, Ahnen DJ, Meester RGS, Barzi A, Jemal A. Colorectal cancer statistics, 2017. *CA Cancer J Clin*. 2017;67:177-193. doi: 10.3322/caac.21395

75. American Cancer Society. Colorectal Cancer Facts & Figures 2017-2019

In: Atlanta: American Cancer Society; 2019.

76. U.S. Cancer Statistics Working Group. U.S. Cancer Statistics Data Visualizations Tool, based on November 2018 submission data (1999-2016): [www.cdc.gov/cancer/dataviz](file:///C:\Users\dtarn\Box\UCLA%20AWV%20R01\Protocol%20and%20MOP\www.cdc.gov\cancer\dataviz), June 2019. <https://gis.cdc.gov/Cancer/USCS/DataViz.html>. 2019. Accessed August 1.

77. American Cancer Society. Colorectal Cancer Facts & Figures 2020-2022. 2022.

78. Rao SR, Breen N, Graubard BI. Trends in Black-White Disparities in Breast and Colorectal Cancer Screening Rates in a Changing Screening Environment: The Peters-Belson Approach Using United States National Health Interview Surveys 2000-2010. *Med Care*. 2016;54:133-139. doi: 10.1097/mlr.0000000000000450

79. Ahmed NU, Pelletier V, Winter K, Albatineh AN. Factors explaining racial/ethnic disparities in rates of physician recommendation for colorectal cancer screening. *Am J Public Health*. 2013;103:e91-99. doi: 10.2105/ajph.2012.301034

80. Ananthakrishnan AN, Schellhase KG, Sparapani RA, Laud PW, Neuner JM. Disparities in colon cancer screening in the Medicare population. *Arch Intern Med*. 2007;167:258-264. doi: 10.1001/archinte.167.3.258

81. National Cancer Institute. State Cancer Profiles; Dynamic views of cancer statistics for prioritizing cancer control efforts acess the nation. <https://statecancerprofiles.cancer.gov/risk/index.php?topic=colorec&risk=v09&race=02&sex=0&type=risk&sortVariableName=default&sortOrder=default#results>. 2019. Accessed August 1.

82. Kane WJ, Fleming MA, 2nd, Lynch KT, Friel CM, Williams MD, Hedrick TL, Yan G, Hoang SC. Associations of Race, Ethnicity, and Social Determinants of Health with Colorectal Cancer Screening. *Dis Colon Rectum*. 2022. doi: 10.1097/dcr.0000000000002371

83. Katz SJ, Hofer TP. Socioeconomic disparities in preventive care persist despite universal coverage. Breast and cervical cancer screening in Ontario and the United States. *JAMA*. 1994;272:530-534.

84. Sambamoorthi U, McAlpine DD. Racial, ethnic, socioeconomic, and access disparities in the use of preventive services among women. *Preventive Medicine*. 2003;37:475-484. doi: <https://doi.org/10.1016/S0091-7435(03)00172-5>

85. 2016 National Healthcare Quality and Disparities Report. Content last reviewed June 2018. Agency for Healthcare Research and Quality, Rockville, MD. 2016.

86. Centers for Medicare & Medicaid Services - Medicare Learning Center. MLN Booklet. Initial Preventive Physical Examination. <https://www.cms.gov/Outreach-and-Education/Medicare-Learning-Network-MLN/MLNProducts/downloads//MPS_QRI_IPPE001a.pdf>. 2018. Accessed August 7.

87. Centers for Medicare & Medicaid Services - Medicare Learning Center. MLN Booklet. Annual Wellness Visit. <https://www.cms.gov/Outreach-and-Education/Medicare-Learning-Network-MLN/MLNProducts/Downloads/AWV_Chart_ICN905706.pdf>. 2018. Accessed July 16.

88. Centers for Medicare & Medicaid Services - Medicare Learning Center. MLN Booklet. Advance Care Planning. <https://www.cms.gov/Outreach-and-Education/Medicare-Learning-Network-MLN/MLNProducts/Downloads/AdvanceCarePlanning.pdf>. 2018. Accessed August 7.

89. Edelberg C. Advance Care Planning With and Without an Annual Wellness Visit. *ED Manag*. 2016;28:69-71.

90. Goetzel RZ, Staley P, Ogden L, Stange P, Fox J, Spangler J, Tabrizi M, Beckowski M, Kowlessar N, Glasgow RE, et al. A framework for patient-centered health risk assessments - providing health promotion and disease prevention services to Medicare beneficiaries. In: Atlanta, GA: US Department of Health and Human Services, Centers for Disease Control and Prevention. Available at: <https://www.cdc.gov/policy/hst/hra/frameworkforhra.pdf>. Accessed on August 8, 2019; 2011.

91. Beckman AL, Becerra AZ, Marcus A, DuBard CA, Lynch K, Maxson E, Mostashari F, King J. Medicare Annual Wellness Visit association with healthcare quality and costs. *Am J Manag Care*. 2019;25:e76-e82.

92. Jiang M, Hughes DR, Wang W. The effect of Medicare's Annual Wellness Visit on preventive care for the elderly. *Prev Med*. 2018;116:126-133. doi: 10.1016/j.ypmed.2018.08.035

93. Tao G. Utilization pattern of other preventive services during the US Medicare annual wellness visit. *Preventive medicine reports*. 2018;10:210-211. doi: 10.1016/j.pmedr.2017.12.014

94. Shen AK, Warnock R, Selna W, MaCurdy TE, Chu S, Kelman JA. Vaccination among Medicare-fee-for service beneficiaries: Characteristics and predictors of vaccine receipt, 2014-2017. *Vaccine*. 2019;37:1194-1201. doi: 10.1016/j.vaccine.2019.01.010

95. Farford BA, Baggett CL, Paredes Molina CS, Ball CT, Dover CM. Impact of an RN-led Medicare Annual Wellness Visit on Preventive Services in a Family Medicine Practice. *J Appl Gerontol*. 2021;40:865-871. doi: 10.1177/0733464820947928

96. Zorek JA, Subash M, Fike DS, MacLaughlin AA, Young RB, Samiuddin M, MacLaughlin EJ. Impact of an Interprofessional Teaching Clinic on Preventive Care Services. *Fam Med*. 2015;47:558-561.

97. Galvin SL, Grandy R, Woodall T, Parlier AB, Thach S, Landis SE. Improved Utilization of Preventive Services Among Patients Following Team-Based Annual Wellness Visits. *N C Med J*. 2017;78:287-295. doi: 10.18043/ncm.78.5.287

98. Chung S, Lesser LI, Lauderdale DS, Johns NE, Palaniappan LP, Luft HS. Medicare annual preventive care visits: use increased among fee-for-service patients, but many do not participate. *Health Aff (Millwood)*. 2015;34:11-20. doi: 10.1377/hlthaff.2014.0483

99. Palmer MK, Jacobson M, Enguidanos S. Advance Care Planning For Medicare Beneficiaries Increased Substantially, But Prevalence Remained Low. *Health Aff (Millwood)*. 2021;40:613-621. doi: 10.1377/hlthaff.2020.01895

100. Nothelle SK, McGuire M, Boyd CM, Colburn JL. Effects of screening for geriatric conditions and advance care planning at the Medicare Annual Wellness Visit. *J Am Geriatr Soc*. 2022;70:579-584. doi: 10.1111/jgs.17546

101. Ganguli I, Souza J, McWilliams JM, Mehrotra A. Association Of Medicare's Annual Wellness Visit With Cancer Screening, Referrals, Utilization, And Spending. *Health Aff (Millwood)*. 2019;38:1927-1935. doi: 10.1377/hlthaff.2019.00304

102. Misra A, Lloyd JT. Hospital utilization and expenditures among a nationally representative sample of Medicare fee-for-service beneficiaries 2 years after receipt of an Annual Wellness Visit. *Prev Med*. 2019;129:105850. doi: <https://doi.org/10.1016/j.ypmed.2019.105850>

103. Centers for Medicare & Medicaid Services. Medicare Enrollment - National Trends 1966-2013. <https://www.cms.gov/Research-Statistics-Data-and-Systems/Statistics-Trends-and-Reports/MedicareEnrpts/Downloads/SMI2013.pdf>. 2013. Accessed August 7.

104. Centers for Medicare & Medicaid Services. Beneficiaries utilizing free preventive services by state, 2016. <https://downloads.cms.gov/files/Beneficiaries%20Utilizing%20Free%20Preventive%20Services%20by%20State%20YTD%202016.pdf>. 2017. Accessed August 8.

105. U.S. Department of Health and Human Services. Annual Performance Plan and Report: Goal 1. Objective C: Emphasize primary and preventive care, linked with community prevention services. <https://www.hhs.gov/about/budget/performance/goal-1-objective-c/index.html>. 2015. Accessed August 7.

106. Shen AK, Warnock R, Kelman JA. Driving immunization through the Medicare Annual Wellness Visit: A growing opportunity. *Vaccine*. 2017;35:6938-6940. doi: 10.1016/j.vaccine.2017.10.055

107. Lind KE, Hildreth K, Lindrooth R, Crane LA, Morrato E, Perraillon MC. Ethnoracial Disparities in Medicare Annual Wellness Visit Utilization: Evidence From a Nationally Representative Database. *Med Care*. 2018;56:761-766. doi: 10.1097/MLR.0000000000000962

108. Ganguli I, Souza J, McWilliams JM, Mehrotra A. Trends in Use of the US Medicare Annual Wellness Visit, 2011-2014. *JAMA*. 2017;317:2233-2235. doi: 10.1001/jama.2017.4342

109. Ganguli I, Souza J, McWilliams JM, Mehrotra A. Practices Caring For The Underserved Are Less Likely To Adopt Medicare's Annual Wellness Visit. *Health Aff (Millwood)*. 2018;37:283-291. doi: 10.1377/hlthaff.2017.1130

110. Hu J, Jensen GA, Nerenz D, Tarraf W. Medicare's Annual Wellness Visit in a Large Health Care Organization: Who Is Using It? *Ann Intern Med*. 2015;163:567-568. doi: 10.7326/L15-5145

111. Kandula NR, Wen M, Jacobs EA, Lauderdale DS. Low rates of colorectal, cervical, and breast cancer screening in Asian Americans compared with non-Hispanic whites: Cultural influences or access to care? *Cancer*. 2006;107:184-192. doi: 10.1002/cncr.21968

112. Wang H, Roy S, Kim J, Farazi PA, Siahpush M, Su D. Barriers of colorectal cancer screening in rural USA: a systematic review. *Rural Remote Health*. 2019;19:5181. doi: 10.22605/rrh5181

113. Fiscella K, Franks P, Clancy CM. Skepticism toward medical care and health care utilization. *Med Care*. 1998;36:180-189.

114. McPhee SJ, Bird JA, Davis T, Ha NT, Jenkins CN, Le B. Barriers to breast and cervical cancer screening among Vietnamese-American women. *Am J Prev Med*. 1997;13:205-213.

115. Vedel I, Puts MTE, Monette M, Monette J, Bergman H. Barriers and facilitators to breast and colorectal cancer screening of older adults in primary care: A systematic review. *Journal of Geriatric Oncology*. 2011;2:85-98. doi: <https://doi.org/10.1016/j.jgo.2010.11.003>

116. Dressler J, Johnsen AT, Madsen LJ, Rasmussen M, Jorgensen LN. Factors affecting patient adherence to publicly funded colorectal cancer screening programmes: a systematic review. *Public Health*. 2021;190:67-74. doi: 10.1016/j.puhe.2020.10.025

117. Parente ST, Salkever DS, DaVanzo J. The role of consumer knowledge of insurance benefits in the demand for preventive health care among the elderly. *Health Econ*. 2005;14:25-38. doi: 10.1002/hec.907

118. Scott TL, Gazmararian JA, Williams MV, Baker DW. Health literacy and preventive health care use among Medicare enrollees in a managed care organization. *Med Care*. 2002;40:395-404.

119. Guessous I, Dash C, Lapin P, Doroshenk M, Smith RA, Klabunde CN, National Colorectal Cancer Roundtable Screening Among the 65 Plus Task G. Colorectal cancer screening barriers and facilitators in older persons. *Prev Med*. 2010;50:3-10. doi: 10.1016/j.ypmed.2009.12.005

120. Jacobs EA, Karavolos K, Rathouz PJ, Ferris TG, Powell LH. Limited English proficiency and breast and cervical cancer screening in a multiethnic population. *Am J Public Health*. 2005;95:1410-1416. doi: 10.2105/AJPH.2004.041418

121. Woloshin S, Schwartz LM, Katz SJ, Welch HG. Is language a barrier to the use of preventive services? *J Gen Intern Med*. 1997;12:472-477. doi: 10.1046/j.1525-1497.1997.00085.x

122. Casey MM, Thiede Call K, Klingner JM. Are rural residents less likely to obtain recommended preventive healthcare services? *Am J Prev Med*. 2001;21:182-188.

123. Coughlin SS, Thompson TD. Colorectal cancer screening practices among men and women in rural and nonrural areas of the United States, 1999. *J Rural Health*. 2004;20:118-124.

124. Coughlin SS, Thompson TD, Hall HI, Logan P, Uhler RJ. Breast and cervical carcinoma screening practices among women in rural and nonrural areas of the United States, 1998-1999. *Cancer*. 2002;94:2801-2812. doi: 10.1002/cncr.10577

125. Tetuan TM, Ohm R, Herynk MH, Ebberts M, Wendling T, Mosier MC. The Affordable Health Care Act annual wellness visits: the effectiveness of a nurse-run clinic in promoting adherence to mammogram and colonoscopy recommendations. *J Nurs Adm*. 2014;44:270-275. doi: 10.1097/NNA.0000000000000066

126. Gilbert A, Kanarek N. Colorectal cancer screening: physician recommendation is influential advice to Marylanders. *Prev Med*. 2005;41:367-379. doi: 10.1016/j.ypmed.2005.01.008

127. Klabunde CN, Schenck AP, Davis WW. Barriers to colorectal cancer screening among Medicare consumers. *Am J Prev Med*. 2006;30:313-319. doi: 10.1016/j.amepre.2005.11.006

128. Salloum RG, Kohler RE, Jensen GA, Sheridan SL, Carpenter WR, Biddle AK. U.S. Preventive Services Task Force recommendations and cancer screening among female Medicare beneficiaries. *J Womens Health (Larchmt)*. 2014;23:211-217. doi: 10.1089/jwh.2013.4421

129. Woodall T, Landis SE, Galvin SL, Plaut T, Roth McClurg MT. Provision of annual wellness visits with comprehensive medication management by a clinical pharmacist practitioner. *Am J Health Syst Pharm*. 2017;74:218-223. doi: 10.2146/ajhp150938

130. Warshany K, Sherrill CH, Cavanaugh J, Ives TJ, Shilliday BB. Medicare annual wellness visits conducted by a pharmacist in an internal medicine clinic. *Am J Health Syst Pharm*. 2014;71:44-49. doi: 10.2146/ajhp130202

131. Thomas MH, Goode JV. Development and implementation of a pharmacist-delivered Medicare annual wellness visit at a family practice office. *J Am Pharm Assoc (2003)*. 2014;54:427-434. doi: 10.1331/JAPhA.2014.13218

132. Evans TA, Fabel PH, Ziegler B. Community pharmacist-delivered Medicare Annual Wellness Visits within a family medicine practice. *J Am Pharm Assoc (2003)*. 2017;57:S247-S251. doi: 10.1016/j.japh.2017.02.015

133. Wilson CG, Park I, Sutherland SE, Ray L. Assessing pharmacist-led annual wellness visits: Interventions made and patient and physician satisfaction. *J Am Pharm Assoc (2003)*. 2015;55:449-454. doi: 10.1331/JAPhA.2015.14229

134. Dougherty MK, Brenner AT, Crockett SD, Gupta S, Wheeler SB, Coker-Schwimmer M, Cubillos L, Malo T, Reuland DS. Evaluation of Interventions Intended to Increase Colorectal Cancer Screening Rates in the United States: A Systematic Review and Meta-analysis. *JAMA internal medicine*. 2018;178:1645-1658. doi: 10.1001/jamainternmed.2018.4637

135. Sapkota S, Brien JA, Greenfield JR, Aslani P. A Systematic Review of Interventions Addressing Adherence to Anti-Diabetic Medications in Patients with Type 2 Diabetes--Components of Interventions. *PLoS One*. 2015;10:e0128581. doi: 10.1371/journal.pone.0128581

136. Slater JS, Henly GA, Ha CN, Malone ME, Nyman JA, Diaz S, McGovern PG. Effect of direct mail as a population-based strategy to increase mammography use among low-income underinsured women ages 40 to 64 years. *Cancer Epidemiol Biomarkers Prev*. 2005;14:2346-2352. doi: 10.1158/1055-9965.Epi-05-0034

137. Mohan G, Chattopadhyay SK, Ekwueme DU, Sabatino SA, Okasako-Schmucker DL, Peng Y, Mercer SL, Thota AB. Economics of Multicomponent Interventions to Increase Breast, Cervical, and Colorectal Cancer Screening: A Community Guide Systematic Review. *Am J Prev Med*. 2019;57:557-567. doi: 10.1016/j.amepre.2019.03.006

138. Stone EG, Morton SC, Hulscher ME, Maglione MA, Roth EA, Grimshaw JM, Mittman BS, Rubenstein LV, Rubenstein LZ, Shekelle PG. Interventions That Increase Use of Adult Immunization and Cancer Screening Services. *Ann Intern Med*. 2002;136:641-651. doi: 10.7326/0003-4819-136-9-200205070-00006

139. Community Preventive Services Task Force. Increasing Breast Cancer Screening: Multicomponent Interventions. Community Preventive Services Task Force Finding and Rationale Statement. Ratified August 2016. In; 2016.

140. Walugembe DR, Sibbald S, Le Ber MJ, Kothari A. Sustainability of public health interventions: where are the gaps? *Health Res Policy Syst*. 2019;17:8. doi: 10.1186/s12961-018-0405-y

141. Scheirer MA. Is Sustainability Possible? A Review and Commentary on Empirical Studies of Program Sustainability. *American Journal of Evaluation*. 2005;26:320-347. doi: 10.1177/1098214005278752

142. Greenhalgh T, Robert G, Bate P, Kyriakidou O, Macfarlane F, Peacock R. How to spread good ideas. 2004.

143. Wiltsey Stirman S, Kimberly J, Cook N, Calloway A, Castro F, Charns M. The sustainability of new programs and innovations: a review of the empirical literature and recommendations for future research. *Implementation Science*. 2012;7:17. doi: 10.1186/1748-5908-7-17

144. Johnson K, Collins D, Shamblen S, Kenworthy T, Wandersman A. Long-Term Sustainability of Evidence-Based Prevention Interventions and Community Coalitions Survival: a Five and One-Half Year Follow-up Study. *Prev Sci*. 2017;18:610-621. doi: 10.1007/s11121-017-0784-2

145. Sansano-Nadal O, Giné-Garriga M, Brach JS, Wert DM, Jerez-Roig J, Guerra-Balic M, Oviedo G, Fortuño J, Gómara-Toldrà N, Soto-Bagaria L, et al. Exercise-Based Interventions to Enhance Long-Term Sustainability of Physical Activity in Older Adults: A Systematic Review and Meta-Analysis of Randomized Clinical Trials. *Int J Environ Res Public Health*. 2019;16. doi: 10.3390/ijerph16142527

146. Grohskopf LA, Alyanak E, Broder KR, Walter EB, Fry AM, Jernigan DB. Prevention and Control of Seasonal Influenza with Vaccines: Recommendations of the Advisory Committee on Immunization Practices - United States, 2019-20 Influenza Season. *MMWR Recomm Rep*. 2019;68:1-21. doi: 10.15585/mmwr.rr6803a1

147. Dooling KL, Guo A, Patel M, Lee GM, Moore K, Belongia EA, Harpaz R. Recommendations of the Advisory Committee on Immunization Practices for Use of Herpes Zoster Vaccines. *MMWR Morb Mortal Wkly Rep*. 2018;67:103-108. doi: 10.15585/mmwr.mm6703a5

148. Kobayashi M, Farrar JL, Gierke R, Britton A, Childs L, Leidner AJ, Campos-Outcalt D, Morgan RL, Long SS, Talbot HK, et al. Use of 15-Valent Pneumococcal Conjugate Vaccine and 20-Valent Pneumococcal Conjugate Vaccine Among U.S. Adults: Updated Recommendations of the Advisory Committee on Immunization Practices - United States, 2022. *MMWR Morbidity and mortality weekly report*. 2022;71:109-117. doi: 10.15585/mmwr.mm7104a1

149. Liang JL, Tiwari T, Moro P, Messonnier NE, Reingold A, Sawyer M, Clark TA. Prevention of Pertussis, Tetanus, and Diphtheria with Vaccines in the United States: Recommendations of the Advisory Committee on Immunization Practices (ACIP). *MMWR Recommendations and reports : Morbidity and mortality weekly report Recommendations and reports*. 2018;67:1-44. doi: 10.15585/mmwr.rr6702a1

150. Schillie S, Wester C, Osborne M, Wesolowski L, Ryerson AB. CDC Recommendations for Hepatitis C Screening Among Adults - United States, 2020. *MMWR Recommendations and reports : Morbidity and mortality weekly report Recommendations and reports*. 2020;69:1-17. doi: 10.15585/mmwr.rr6902a1

151. Siu AL, Bibbins-Domingo K, Grossman DC, Baumann LC, Davidson KW, Ebell M, Garcia FA, Gillman M, Herzstein J, Kemper AR, et al. Screening for Depression in Adults: US Preventive Services Task Force Recommendation Statement. *Jama*. 2016;315:380-387. doi: 10.1001/jama.2015.18392

152. U.S. Preventive Services Task Force. Published recommendations. <https://www.uspreventiveservicestaskforce.org/BrowseRec/Index/browse-recommendations>. Accessed September 5.

153. Advisory Committee on Immunization Practices (ACIP). ACIP Recommendations. <https://www.cdc.gov/vaccines/acip/recommendations.html>. Accessed November 2.

154. U.S. Department of Health and Human Services Centers for Disease Control and Prevention. Recommended Adult Immunization Schedule for ages 19 years or older. United States. 2019. <https://www.cdc.gov/vaccines/schedules/downloads/adult/adult-combined-schedule.pdf>. Accessed November 2.

155. Haines TP, Hemming K. Stepped-wedge cluster-randomised trials: level of evidence, feasibility and reporting. *J Physiother*. 2018;64:63-66. doi: 10.1016/j.jphys.2017.11.008

156. Hemming K, Haines TP, Chilton PJ, Girling AJ, Lilford RJ. The stepped wedge cluster randomised trial: rationale, design, analysis, and reporting. *BMJ*. 2015;350:h391. doi: 10.1136/bmj.h391

157. Hussey MA, Hughes JP. Design and analysis of stepped wedge cluster randomized trials. *Contemp Clin Trials*. 2007;28:182-191. doi: 10.1016/j.cct.2006.05.007

158. Tarn DM, Pace WD, Tseng CH, Callen E, Loskutova NY, Stange KC, Wenger NS. Outcomes of A Virtual Practice-Tailored Medicare Annual Wellness Visit Intervention. *Journal of the American Board of Family Medicine : JABFM*. 2023;36:501-509. doi: 10.3122/jabfm.2022.220342R1

159. Cohen D, McDaniel RR, Jr., Crabtree BF, Ruhe MC, Weyer SM, Tallia A, Miller WL, Goodwin MA, Nutting P, Solberg LI, et al. A practice change model for quality improvement in primary care practice. *J Healthc Manag*. 2004;49:155-168; discussion 169-170.

160. Miller WL, Rubinstein EB, Howard J, Crabtree BF. Shifting Implementation Science Theory to Empower Primary Care Practices. *Ann Fam Med*. 2019;17:250-256. doi: 10.1370/afm.2353

161. Tarn DM, Guzman JR, Good JS, Wenger NS, Coulter ID, Paterniti DA. Provider and patient expectations for dietary supplement discussions. *J Gen Intern Med*. 2014;29:1242-1249. doi: 10.1007/s11606-014-2899-5

162. Broglio K. Randomization in Clinical Trials: Permuted Blocks and Stratification. *JAMA*. 2018;319:2223-2224. doi: 10.1001/jama.2018.6360

163. Maleyeff L, Li F, Haneuse S, Wang R. Assessing exposure-time treatment effect heterogeneity in stepped-wedge cluster randomized trials. *Biometrics*. 2022. doi: 10.1111/biom.13803

164. Glasgow RE, Harden SM, Gaglio B, Rabin B, Smith ML, Porter GC, Ory MG, Estabrooks PA. RE-AIM Planning and Evaluation Framework: Adapting to New Science and Practice With a 20-Year Review. *Frontiers in Public Health*. 2019;7. doi: 10.3389/fpubh.2019.00064

165. Glasgow RE, Vogt TM, Boles SM. Evaluating the public health impact of health promotion interventions: the RE-AIM framework. *Am J Public Health*. 1999;89:1322-1327. doi: 10.2105/ajph.89.9.1322

166. Holtrop JS, Rabin BA, Glasgow RE. Qualitative approaches to use of the RE-AIM framework: rationale and methods. *BMC Health Serv Res*. 2018;18:177-177. doi: 10.1186/s12913-018-2938-8

167. Gaglio B, Shoup JA, Glasgow RE. The RE-AIM framework: a systematic review of use over time. *American journal of public health*. 2013;103:e38-46. doi: 10.2105/ajph.2013.301299

168. Crabtree BF MW, Crabtree BF, Miller WL. *Using Codes and Code Manuals* Sage Publications; 1999.

169. Miles MB HA, Saldaña J. *Qualitative data analysis : a methods sourcebook*. Third edition. ed. ed. SAGE Publications, Inc; 2014.
